# High expression of N-myc (and STAT) interactor predicts poor prognosis and promotes tumor growth in human glioblastoma

**DOI:** 10.18632/oncotarget.3208

**Published:** 2014-12-30

**Authors:** Delong Meng, Yuanyuan Chen, Dapeng Yun, Yingjie Zhao, Jingkun Wang, Tao Xu, Xiaoying Li, Yuqi Wang, Li Yuan, Ruochuan Sun, Xiao Song, Cong Huai, Lingna Hu, Song Yang, Taishan Min, Juxiang Chen, Hongyan Chen, Daru Lu

**Affiliations:** ^1^ State Key Laboratory of Genetic Engineering and MOE Key Laboratory of Contemporary Anthropology, Collaborative Innovation Center for Genetics and Development, Institute of Genetics, School of Life Sciences, Fudan University, Shanghai, China; ^2^ Department of Neurosurgery, Shanghai Institute of Neurosurgery, Changzheng Hospital, Second Military Medical University, Shanghai, China; ^3^ The Eighth Department of General Surgery and Department of Pathology, The First Affiliated Hospital of Anhui Medical University, Hefei, China

**Keywords:** NMI, glioblastoma, prognosis, immunohistochemistry, TCGA

## Abstract

Glioma is the most malignant brain tumor and glioblastoma (GBM) is the most aggressive type. The involvement of N-myc (and STAT) interactor (NMI) in tumorigenesis was sporadically reported but far from elucidation. This study aims to investigate roles of NMI in human glioma. Three independent cohorts, the Chinese tissue microarray (TMA) cohort (N = 209), the Repository for Molecular Brain Neoplasia Data (Rembrandt) cohort (N = 371) and The Cancer Genome Atlas (TCGA) cohort (N = 528 or 396) were employed. Transcriptional or protein levels of NMI expression were significantly increased according to tumor grade in all three cohorts. High expression of NMI predicted significantly unfavorable clinical outcome for GBM patients, which was further determined as an independent prognostic factor. Additionally, expression and prognostic value of *NMI* were associated with molecular features of GBM including *PTEN* deletion and *EGFR* amplification in TCGA cohort. Furthermore, overexpression or depletion of NMI revealed its regulation on G1/S progression and cell proliferation (both *in vitro* and *in vivo*), and this effect was partially dependent on STAT1, which interacted with and was regulated by NMI. These data demonstrate that NMI may serve as a novel prognostic biomarker and a potential therapeutic target for glioblastoma.

## INTRODUCTION

Glioma, accounting for approximately 30% of all primary brain and central nervous system (CNS) tumors and 80% of malignant primary brain and CNS tumors [1], is often fast growing with poor prognosis for the patients. The characteristics of glioma including complex cellular composition, diffuse invasiveness and ability to evade therapies have puzzled researchers for decades and impeded progress of effective treatments [2]. Among gliomas, glioblastoma multiforme (GBM) is the most common and malignant type, with median overall survival of about 15–16 months [3] and 5-year survival rate less than 5% [1, 4]. Despite multimodal therapies including maximal resection followed by adjuvant chemotherapy and radiotherapy [5], the clinical outcome of GBM patients remains dismal [6, 7]. Thus, valuable prognostic biomarkers and potential molecular targets for GBMs have been recently studied [8-12] and are still urgently desired, and the underlying mechanisms remain to be elucidated.

Genetic alterations of several important genes may contribute to the pathogenesis of GBM and differ from patient to patient. Therefore, personalized treatment regimens may be more effective for patients. Recently, substantial efforts have been made in the identification of molecular subtypes [13, 14] and biomarkers associated with GBM patients' survival [15-17], to better understand the pathogenesis of GBM. Several public resources, such as the Repository of Molecular Brain Neoplasia Data (Rembrandt) database [18] and The Cancer Genome Atlas (TCGA) network [19] have provided insight into the molecular carcinogenesis of GBM, affording opportunities for researchers to correlate gene expression with multidimensional clinical and molecular features of patients [15, 16, 20-22]. Based on gene expression studies of GBM tissues, TCGA network identified several distinct molecular subtypes of GBM, including classical, mesenchymal, proneural and neural [23]. Subsequently, another subgroup called glioma-CpG island methylator phenotype (G-CIMP), which belonged to the proneural subtype and was tightly associated with *IDH1* mutation, was further identified with significant survival benefit for patients [24]. Thus, uncovering new molecular targets and prognostic factors, and revealing the correlation of their expression with molecular features of GBM, may provide chances to improve the clinical outcome of GBM patients.

N-myc (and STAT) interactor (NMI), was first identified in 1996 as an interactor of N-myc and C-myc oncogenes using a yeast genetic screen [25], and further reported to interact with several members of STATs and potentiate JAK/STAT pathway [26], which has been demonstrated to participate in the development, proliferation, invasiveness, inflammation, metastasis, immune regulation and microenvironment of tumors [27]. Moreover, recent studies have revealed interactions of NMI with a variety of proteins including BRCA1, Sox10, IFI35, CKIP-1, Tip60, ARF, FMDV 2C, IRF7, IRE1α and Hsp105β [28-37], with several of them suggesting the involvement of NMI in cytokine response and virus-related cellular process [33-35]. However, the function of NMI, particularly its potential role in tumorigenesis, has not been well characterized. Previous studies showed that NMI was highly expressed in myeloid leukemias and pancreatic ductal adenocarcinomas [25, 38]. By contrast, it was reported recently that NMI retarded breast cancer growth [39] while loss of NMI promoted epithelial-mesenchymal transition of breast cancer [40, 41]. However, the function and prognostic significance of NMI in glioma have never been studied.

In this study, we first investigated protein expression of NMI in the Chinese glioma cohort using a tissue microarray (TMA), and validated its mRNA expression in a subset of this cohort and another two independent cohorts, the Rembrandt cohort and the TCGA cohort. We next estimated the clinical significance of NMI as an independent prognostic factor in all three cohorts. Expression and clinical significance of *NMI* were also analyzed according to molecular features of GBM in the TCGA cohort. Finally, we explored the functions and potential molecular mechanisms of NMI in tumor growth. Our data demonstrate NMI as a novel independent prognostic factor for GBM patients and highlight an important role of NMI in tumorigenesis and progression of GBM.

## RESULTS

### NMI is elevated in human gliomas

**Figure 1:**
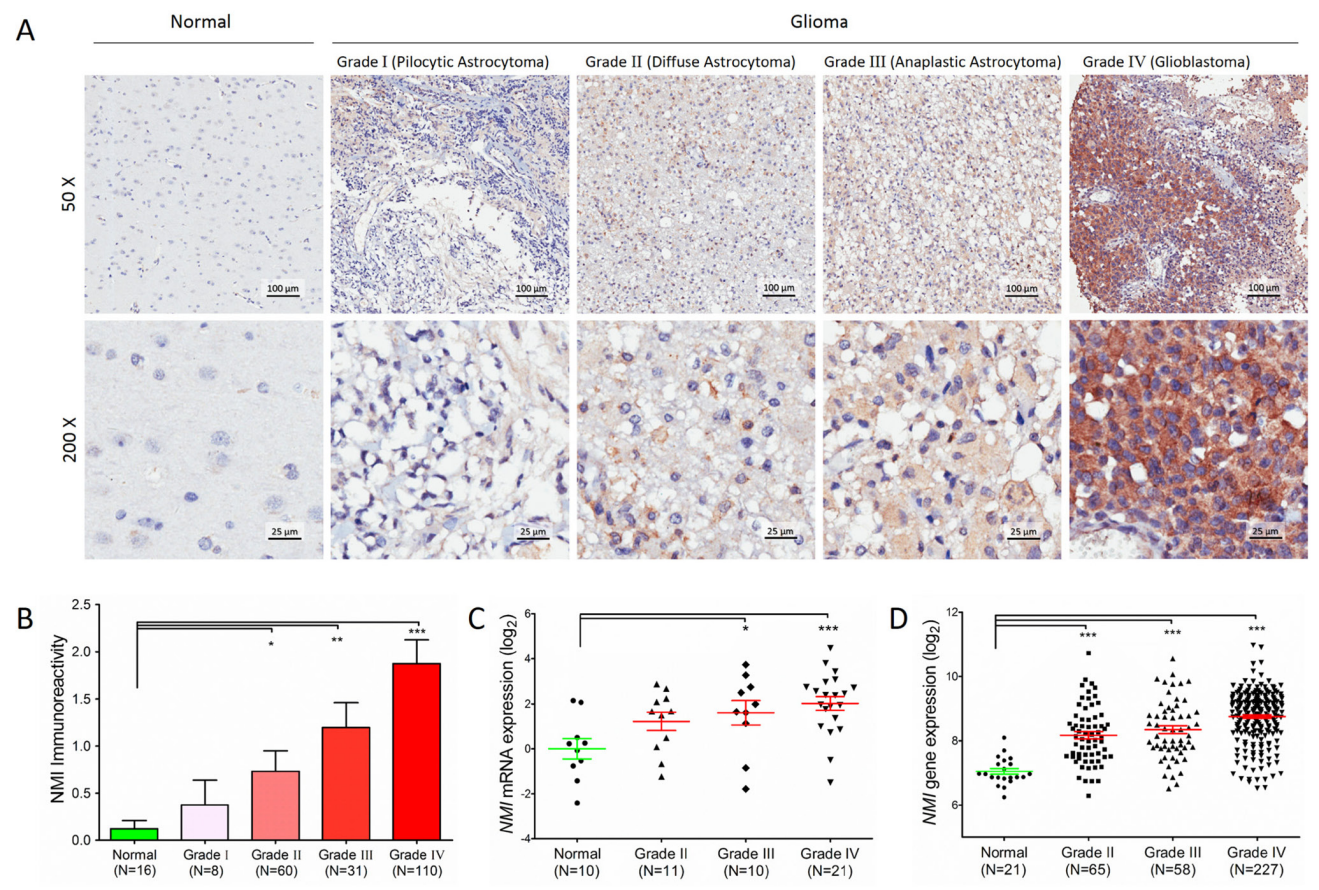
Protein expression and mRNA transcript of*NMI* gene are elevated in human gliomas (A) NMI protein expression was analyzed by immunohistochemistry staining and representative staining images in human glioma of different WHO grades and normal brain specimens were shown as indicated. Magnification: ×50, upper; ×200, lower. Scale bars: 100 μm, upper; 25 μm, lower. (B) Immunoreactivity scores of NMI staining in normal brain samples and WHO grade I to grade IV gliomas are represented as mean ± SEM. (C) *NMI* mRNA expression was analyzed by real time RT-PCR assays in human glioma of different WHO grades and normal brain tissues, and *GADPH* was used as an internal control. The value represents log2 of gene expression level of samples. (D) *NMI* gene expression was compared between glioma of different WHO grades and normal tissues in the Rembrandt cohort. A single spot represents the *NMI* expression value (log 2 scale) of an individual patient, with a line in the middle representing the median expression value and error bars representing the SEM. Statistical differences between normal tissues and gliomas of different WHO grades were determined using two tailed student's t-test. *, *P*<0.05; **, *P*<0.01; ***, *P*<0.001.

In total, 209 cases of human glioma patients were enrolled in the Chinese TMA cohort, including 8 pilocytic astrocytomas (grade I), 60 diffuse astrocytomas (grade II), 31 anaplastic astrocytomas (grade III) and 110 glioblastomas (GBM, grade IV) according to the WHO grading schedule, and a tissue microarray (TMA) was constructed. As shown in Table 1, the patients' median overall survival was 21 months for all gliomas and 12 months for GBMs. The expression levels of NMI protein were investigated by immunohistochemical staining performed on the TMA. We found that NMI was mainly expressed in cytoplasm of cells. Compared to 16 cases of normal tissues, the immunoreactivity of NMI was unequivocally elevated in human gliomas and increased according to WHO grades, while the highest expression of NMI was observed in GBM samples (Figure 1A and 1B, Table 1).

**Table 1:** The clinicopathologic characteristics of the 209 glioma patients in the Chinese TMA cohort

Characteristics	All glioma (N=209)	WHO grade			
		Grade I (N=8)	Grade II (N=60)	Grade III (N=31)	Grade IV (N=110)
Gender					
Male	144	5(62.5%)	47(78.3%)	18(58.1%)	74(67.3%)
Female	65	3(37.5%)	13(21.7%)	13(41.9%)	36(32.7%)
Age at diagnosis (year)					
< 60	173	7(87.5%)	57(95.0%)	26(83.9%)	83(75.5%)
≥60	36	1(12.5%)	3(5.0%)	5(16.1%)	27(24.5%)
Tumor origin					
Primary	183	8(100%)	56(93.3%)	23(74.2%)	96(87.3%)
Secondary	26	0(0.0%)	4(6.7%)	8(25.8%)	14(12.7%)
Extend of resection					
Gross total	164	6(75.0%)	49(81.7%)	25(80.6%)	84(76.4%)
Partial	45	2(25.0%)	11(18.3%)	6(19.4%)	26(23.6%)
NMI expression					
Low	175	8(100.0%)	55(91.7%)	26(83.9%)	86(78.2%)
High	34	0(0.0%)	5(8.3%)	5(16.1%)	24(21.8%)
OS (month)					
median (95% CI)	21(17.36-24.64)	NA^a^	73(56.57-89.43)	25(15.17-34.83)	12(10.45-13.55)
PFS (month)					
median (95% CI)	19(15.36-22.64)	NA^a^	73(39.84-106.16)	25(18.25-31.75)	10(7.66-12.34)

Abbreviations: OS, overall survival; PFS, progression-free survival; CI, confidence interval.

a NA, not available due to a large proportion of censored cases.

We further addressed whether the *NMI* gene was also augmented at the transcriptional level. Total RNA was extracted from a subset of 42 human glioma specimens (11 diffuse astrocytomas, 10 anaplastic astrocytomas and 21 glioblastomas) and 10 controls randomly selected from this cohort and subjected to real-time quantitative RT-PCR assays. The mRNA expression of *NMI* was also significantly elevated in human gliomas and increased according to WHO grades (Figure 1C), which was further validated in an independent cohort with more samples, namely the Rembrandt [18] cohort (Figure 1D). Together, these results suggest that NMI is elevated in human gliomas.

### *NMI* expression is correlated with several molecular features of GBM in the TCGA cohort

**Figure 2:**
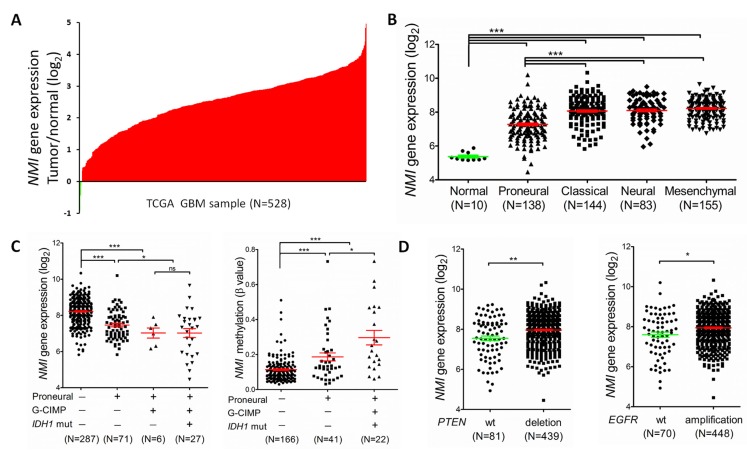
*NMI* expression levels are augmented in GBMs of the TCGA cohort (the Affymetrix platform) and associated with several molecular features (A) *NMI* mRNA expression levels were detected in 528 clinical GBM specimens and 10 cases of normal control tissue obtained by TCGA. The value represents log2 of gene expression level of each GBM sample to the average mRNA of 10 normal samples. The red samples (>0) indicate that the mRNA levels of these GBM tissues were higher than the average of normal brain tissues while the green bars (<0) represent GBM sample with lower *NMI* mRNA expression compared to normal tissues. (B) *NMI* mRNA expression levels were compared between normal samples and different molecular subtypes of GBMs as indicated. (C) *NMI* expression was compared according to subtype (proneural or not), Glioma-CpG Island Methylation Phenotype (G-CIMP) and *IDH1* mutation status (left panel), and *NMI* methylation levels (presented as β values) were analyzed correspondingly (right panel). (D) *NMI* expression was compared according to status of *PTEN* (left panel) or *EGFR* (right panel) mutation as indicated. In these scatter plots (B-D), a single spot represents the *NMI* expression value (log 2 scale) or methylation level (β value) of an individual patient, with a line in the middle representing the median expression value and error bars representing the SEM. Statistical differences were determined by two tailed student's t-test. *, *P*<0.05; **, *P*<0.01; ***, *P*<0.001; ns, not significant.

We next examined the expression profile of the *NMI* gene in another independent cohort with a larger sample size, i.e. the TCGA [23] cohort. Consequently, mRNA expression of *NMI* was discovered to have more than 2 fold up-regulation in over 90% (499/528 for the Affymetrix platform and 364/396 for the Agilent platform) of GBMs compared to the normal controls (Figure 2A and Supplementary Figure S1A). For better understanding of the significance of *NMI* expression in GBMs, a correlation analysis was further conducted between *NMI* expression and molecular features of GBMs. TCGA network has discovered a robust molecular classification of GBMs based on gene expression, which classified GBMs into 4 different subtypes, namely classical, mesenchymal, neural, and proneural [23]. Thus, we screened *NMI* expression according to distinct molecular subtypes of GBM and observed dramatically decreased *NMI* expression in the proneural subtype compared with other three subtypes, while *NMI* expression of each subtype was still evidently higher than that of the normal controls (Figure 2B and Supplementary Figure S1B).

TCGA network has further identified a distinct subgroup of GBM called a glioma-CpG island methylator phenotype (G-CIMP), which belonged to the proneural subgroup and was tightly associated with *IDH1* somatic mutation [24]. Thus, we next explored *NMI* expression according to molecular subgroup (proneural or non-proneural, G-CIMP or non-G-CIMP) and status of *IDH1* mutation. *NMI* expression in patients of the G-CIMP subgroup or with *IDH1* mutation was found to be significantly lower than non-G-CIMP or *IDH1* wild-type patients (Supplementary Table S1). As shown in Figure 2C (left panel), in the G-CIMP subgroup, there was no significant difference of *NMI* expression in patients with or without *IDH1* mutation. However, in the proneural subgroup, there was a dramatic reduction of *NMI* expression in patients of the G-CIMP subgroup compared with non-G-CIMP patients (Figure 2C and Supplementary Figure S1C). Moreover, it was noteworthy that among the non-G-CIMP subgroup, patients of the proneural subtype expressed significantly lower *NMI*. Given that samples of G-CIMP displayed concerted hypermethylation at a large number of loci [24], we asked whether the expression mode of *NMI* according to molecular subtypes of GBM was correlated with its methylation level. Interestingly, as shown in Figure 2C (right panel), among the non-G-CIMP subgroup, *NMI* methylation level was significantly higher in the proneural subtype compared with non-proneural subtypes. Furthermore, among the proneural subtype, G-CIMP patients had further increased methylation level of *NMI* compared with the non-G-CIMP subgroup. Additionally, the reverse correlation of *NMI* expression and its methylation level was consistently observed (Supplementary Figure S1D).

TCGA analyses of GBM have identified several critical genetic aberrations, including mutations in *TP53*, *PTEN*, *NF1*, *EGFR*, *RB1*, *PIK3R1*, *IDH1*, *PIK3CA*, *SPTA1*, *ATRX*, *KEL*, *GABRA6*, *LZTR1*, *CTNND2*, *BRAF*, amplifications of *EGFR*, *CDK4*, *PDGFRA*, *MDM2*, *MET*, *MDM4*, *CDK6*, *MYCN*, *CCND2*, *PIK3CA*, *AKT3*, and deletions of *CDKN2A*, *CDKN2B*, *PTEN*, *CDKN2C*, *RB1*, *PARK2* and *NF1* [19, 42]. To further explore the expression profile of the *NMI* gene, we examined the association between its expression and these common genetic alterations in GBM. Consequently, we found that besides *IDH1* mutation, *NMI* expression was also significantly associated with *PTEN* deletion and *EGFR* amplification (Figure 2D) and Supplementary Figure S1E), *PDGFRA* amplification, *RB1* mutation and *TP53* mutation in both platforms (Supplementary Table S1).

### NMI serves as an independent prognostic factor for GBM patients

**Figure 3:**
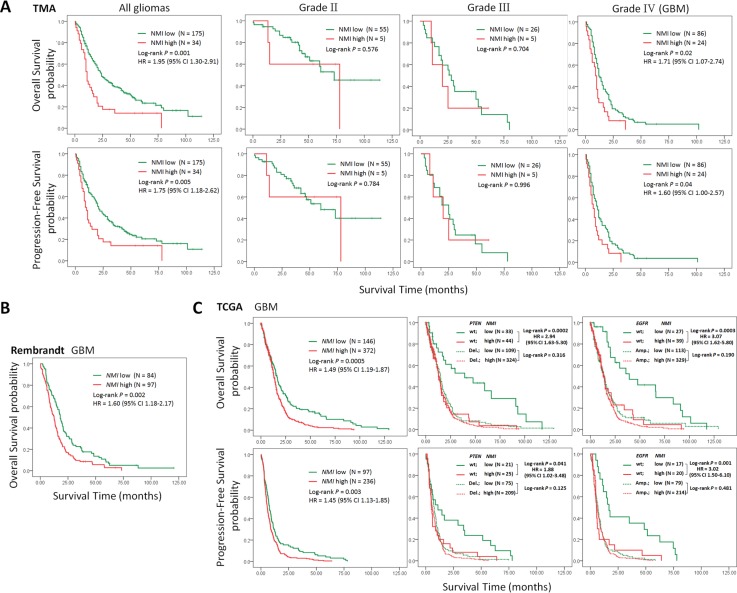
High expression of NMI predicts a poor clinical outcome in human gliomas (A) Kaplan-Meier survival curves were plotted according to different NMI immunoreactivity level for overall survival (OS, upper panels) and progression-free survival (PFS, lower panels) of all glioma patients and patients of different WHO grades in the Chinese TMA cohort. (B) Kaplan-Meier curve was plotted according to different *NMI* gene expression for overall survival of GBM patients in the Rembrandt cohort. (C) Kaplan-Meier plots were estimated according to different *NMI* gene expression for overall survival (upper panels) and progression-free survival (lower panels) of all GBM patients (left panels), or considering the mutation status of *PTEN* (center panels) or *EGFR* (right panels) simultaneously, in the TCGA cohort (Affymetrix platform). *P* values were obtained from log-rank test, while hazard ratio (HR) and 95% confidence interval (CI) were determined by univariate Cox regression model.

To investigate the relationship between NMI expression and clinical prognosis, we first analyzed the prognostic significance of NMI expression in the Chinese TMA cohort. Kaplan-Meier analyses showed that in all glioma patients, high NMI expressers had significantly shorter overall survival (OS) and progression-free survival (PFS; Figure 3A, left panels) than those with low NMI expression. As higher tumor grade is an indubitable risk factor for glioma, we further did similar analyses in patients of each WHO grade (except grade I due to insufficient sample size). As shown in Figure 3A (center panels), NMI expression did not affect clinical outcome for grade II and III glioma patients. However, high NMI expression could predict significantly unfavorable OS and PFS for patients with GBM (Figure 3A, right panels).

Moreover, subsequent univariate and multivariate Cox regression analyses were further conducted to determine the independence of the prognostic value of NMI. After correction for clinical characteristics suggested to be significant prognostic factors in the univariate Cox regression, high NMI expression was an independent risk predictor of both OS (*P* = 0.011, HR = 1.72, 95% CI = 1.13-2.60) and PFS (*P* = 0.048, HR = 1.51, 95% CI = 1.00-2.28) for all glioma patients (Table 2). In addition, high NMI expression could also be an independent risk factor for OS (*P* = 0.025, HR = 1.71, 95% CI = 1.07-2.74) and PFS (*P* = 0.049, HR = 1.60, 95% CI = 1.00-2.57) of GBM patients (Table 2).

**Table 2:** Univariate and multivariate Cox regression of NMI immunoreactivity for overall survival and progression-free survival in all glioma patients and GBM patients in the Chinese TMA cohort

Characteristics	Univariate Cox Regression		Multivariate Cox Regression	
	*P*	HR (95% CI)	*P*	HR (95% CI)
**All glioma (N=209)**				
**OS**				
Gender (male vs. female)	0.276			
Age (≥60 vs. <60)	**3.94E-04**	2.02(1.37-2.98)	0.103	
Tumor origin (Secondary vs. Primary)	0.604			
Resection (gross total vs. patial)	**0.030**	0.66(0.45-0.96)	**0.008**	0.59(0.40-0.87)
Grade (III+IV vs. I+II)	**1.73E-14**	5.93(3.76-9.34)	**2.89E-13**	5.67(3.56-9.03)
NMI immunoreactivity (high vs. low)	**0.001**	1.95(1.30-2.91)	**0.011**	1.72(1.13-2.60)
**PFS**				
Gender (male vs. female)	0.200			
Age (≥60 vs. <60)	**0.001**	1.98(1.35-2.92)	0.106	
Tumor origin (Secondary vs. Primary)	0.151			
Resection (gross total vs. patial)	**0.020**	0.65(0.45-0.93)	**0.012**	0.62(0.43-0.90)
Grade (III+IV vs. I+II)	**1.14E-14**	5.41(3.52-8.30)	**5.84E-13**	5.02(3.24-7.79)
NMI immunoreactivity (high vs. low)	**0.006**	1.76(1.18-2.62)	**0.048**	1.51(1.00-2.28)
**GBM (N=110)**				
**OS**				
Gender (male vs. female)	0.426			
Age (≥60 vs. <60)	0.084			
Tumor origin (Secondary vs. Primary)	0.852			
Resection (gross total vs. patial)	0.966			
NMI immunoreactivity (high vs. low)	**0.025**	1.71(1.07-2.74)	**0.025**	1.71(1.07-2.74)
**PFS**				
Gender (male vs. female)	0.138			
Age (≥60 vs. <60)	0.088			
Tumor origin (Secondary vs. Primary)	0.283			
Resection (gross total vs. patial)	0.913			
NMI immunoreactivity (high vs. low)	**0.049**	1.60(1.00-2.57)	**0.049**	1.60(1.00-2.57)

Abbreviations: OS, overall survival; PFS, progression-free survival; CI, confidence interval; HR, hazard ratio.

Similar analyses conducted in the Rembrandt cohort revealed that, high *NMI* mRNA expression could significantly predict a worse OS for GBM patients in comparison with low *NMI* expression (Figure 3B), which could also serve as an independent prognostic factor in a multivariate Cox regression model (*P* = 0.002, HR = 1.69, 95% CI = 1.21-2.37; Table 3).

**Table 3:** Univariate and multivariate Cox regression of *NMI* expression for overall survival in GBM patients of the Rembrandt cohort

Characteristics	Univariate Cox Regression		Multivariate Cox Regression	
	*P*	HR (95% CI)	*P*	HR (95% CI)
Gender (male vs. female)	0.522			
Age (≥60 vs. <60)	**6.12E-06**	2.23(1.58-3.15)	**1.92E-06**	2.34(1.65-3.32)
*NMI* expression (high vs. low)	**0.003**	1.60(1.18-2.17)	**0.002**	1.69(1.21-2.37)

Abbreviations: HR, hazard ratio; CI, confidence interval

We further explored the correlation between *NMI* expression and GBM patients' clinical outcome in the TCGA cohort. High *NMI* expression was validated to be a unfavorable predictor for patients' OS and PFS (Figure 3C and Supplementary Figure S1F, left panels). We next carried out Kaplan-Meier survival analyses stratified by the status of common genetic aberrations of GBM. As a consequence, the effect of *NMI* on patients' prognosis was correlated with several molecular features including *PTEN* deletion, *PDGFRA* amplification, *PARK2* deletion, *EGFR* amplification, *RB1* deletion and *CDK6* amplification (Supplementary Table S2 and S3). It was rather remarkable that, as shown in Figure 3C (center and right panels), the prognostic significance of *NMI* was highly pronounced in individuals with wild-type *PTEN* (OS, log-rank *P* < 0.001, HR = 2.94, 95% CI = 1.63-5.30; PFS, log-rank *P* = 0.041, HR = 1.88, 95% CI = 1.02-3.48) or *EGFR* (OS, log-rank *P* < 0.001, HR = 3.07, 95% CI = 1.62-5.80; PFS, log-rank *P* = 0.001, HR = 3.02, 95% CI = 1.50-6.10), but disappeared in *PTEN* deleted or *EGFR* amplified individuals in the Affymetrix platform, and similar trends were observed in another platform (Supplementary Figure S1F). In addition, we performed survival analysis stratified by the status of *MGMT* methylation, an important prognostic factor for GBM [5]. However, the difference of *NMI*'s effect on patients' overall survival between *MGMT* unmethylated and methylated patients was only observed in one platform (Supplementary Table S2 and S3).

Multivariate Cox regression further confirmed the prognostic value of *NMI* as an independent predictor for GBM patient's OS (the Affymetrix platform, *P* = 0.021, HR = 1.54, 95% CI = 1.07-2.23; the Agilent platform, *P* = 0.034, HR = 1.78, 95% CI = 1.05-3.04) and PFS (the Affymetrix platform, *P* = 0.033, HR = 1.55, 95% CI = 1.04-2.32; the Agilent platform, *P* = 0.017, HR = 2.10, 95% CI = 1.14-3.86) in the TCGA cohort, after adjusting for clinical characteristics and molecular features (Table 4 and Supplementary Table S4). Taken together, these results indicate that NMI could be an independent prognostic factor of human glioblastoma.

**Table 4:** Univariate and multivariate Cox regression of *NMI* expression for overall survival and progression-free survival in GBM patients of the TCGA cohort (the Affymetrix platform)

Characteristics	Univariate Cox Regression		Multivariate Cox Regression	
	*P*	HR (95% CI)	*P*	HR (95% CI)
**OS**				
Gender (male vs. female)	0.464			
Age (≥60 vs. <60)	**1.42E-10**	1.94(1.58-2.37)	**3.97E-04**	1.72(1.28-2.33)
Tumor origin (Secondary vs. Primary)	**4.42E-05**	0.39(0.25-0.62)	**0.001**	0.39(0.22-0.69)
*MGMT* (methylated vs. unmethylated)	**0.002**	0.67(0.52-0.87)	**0.025**	0.72(0.54-0.96)
G-CIMP (positive vs. negative)	**3.39E-08**	0.32(0.21-0.48)	0.177	
Subtype (proneural vs. non-proneural)	**0.038**	0.79(0.63-0.99)	0.124	
*PTEN* (deletion vs. wild type)	**0.001**	1.64(1.23-2.18)	0.149	
*EGFR* (amplification vs. wild type)	**1.07E-04**	1.85(1.35-2.52)	0.252	
*PARK2* (deletion vs. wild type)	0.529			
*PDGFRA* (amplification vs. wild type)	0.287			
*CDK6* (amplification vs. wild type)	**0.002**	1.50(1.16-1.92)	0.223	
*RB1* (deletion vs. wild type)	0.953			
*IDH1* (mutation vs. wild type)	**1.00E-04**	0.37(0.23-0.61)	0.783	
*NMI* expression (high vs. low)	**0.001**	1.49(1.19-1.87)	**0.021**	1.54(1.07-2.23)
**PFS**				
Gender (male vs. female)	0.35			
Age (≥60 vs. <60)	**7.77E-05**	1.57(1.25-1.96)	0.489	
Tumor origin (Secondary vs. Primary)	**1.82E-06**	0.29(0.18-0.49)	**7.74E-06**	0.21(0.10-0.41)
*MGMT* (methylated vs. unmethylated)	**0.025**	0.73(0.55-0.96)	**0.008**	0.65(0.47-0.89)
G-CIMP (positive vs. negative)	**4.30E-07**	0.29(0.18-0.47)	0.255	
Subtype (proneural vs. non-proneural)	**0.038**	0.76(0.59-0.99)	**0.045**	1.58(1.01-2.45)
*PTEN* (deletion vs. wild type)	**0.003**	1.64(1.18-2.29)	0.698	
*EGFR* (amplification vs. wild type)	**4.56E-05**	2.20(1.51-3.21)	0.281	
*PARK2* (deletion vs. wild type)	0.743			
*PDGFRA* (amplification vs. wild type)	0.807			
*CDK6* (amplification vs. wild type)	**0.025**	1.38(1.04-1.82)	0.358	
*RB1* (deletion vs. wild type)	0.97			
*IDH1* (mutation vs. wild type)	**0.015**	0.50(0.29-0.87)	0.26	
*NMI* expression (high vs. low)	**0.003**	1.45(1.13-1.85)	**0.033**	1.55(1.04-2.32)

Abbreviations: G-CIMP, Glioma-CpG Island Methylator Phenotype; OS, overall survival; PFS, progression-free survival; HR, hazard ratio; CI, confidence interval.

### NMI regulates glioma proliferation *in vitro*

**Figure 4:**
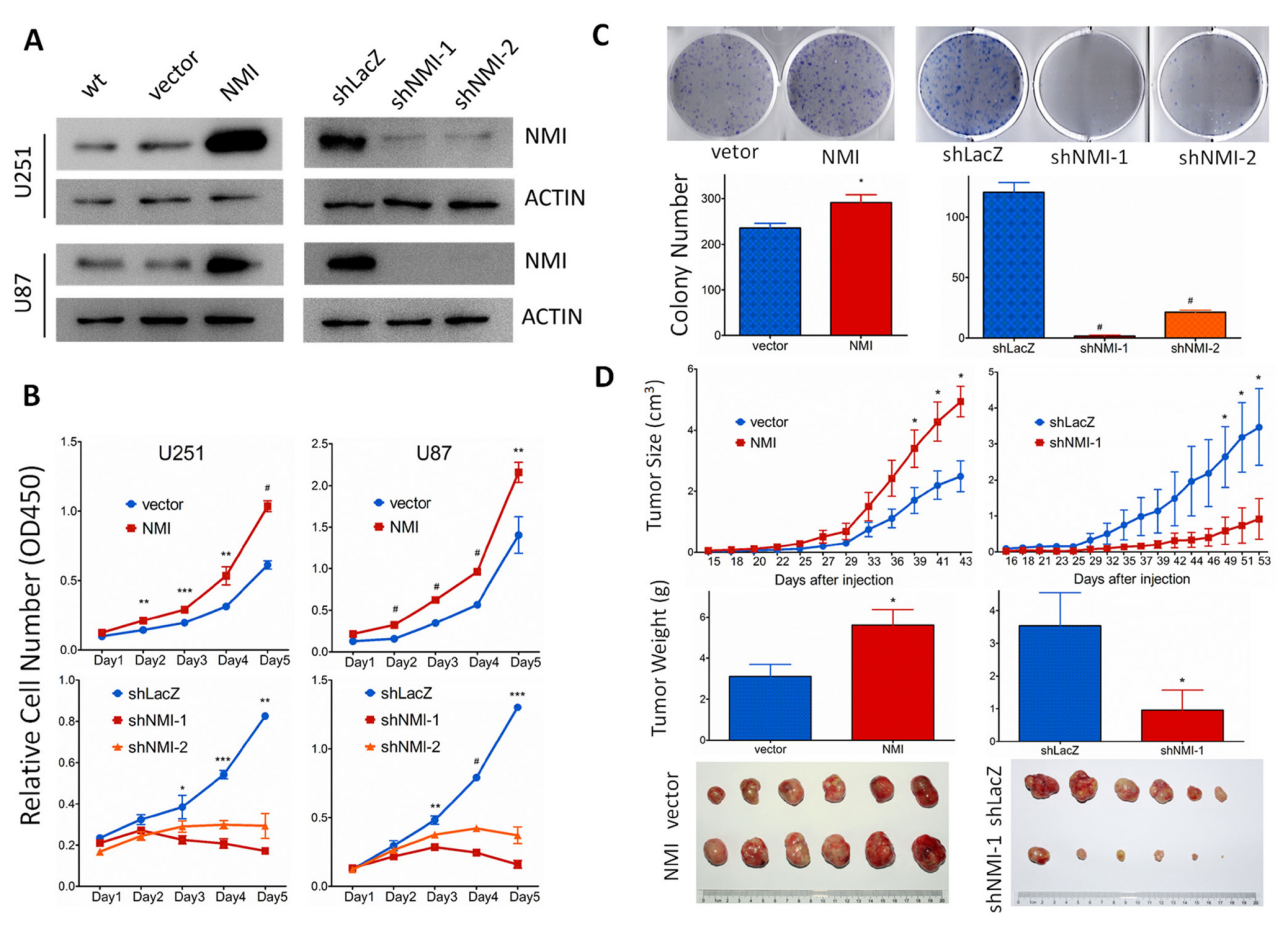
NMI promotes glioma cell growth *in vitro* and *in vivo* (A) Lentivirus-delivered overexpression and knock-down of NMI in U251 and U87 human glioma cell lines were validated by western blot analysis. Two independent shRNAs were used to stably knock down expression of NMI. ACTIN served as a loading control. (B) The cell growth curve of NMI overexpression and knockdown cells was determined by CCK-8 assay. (C) The indefinite proliferation ability of NMI overexpression and knockdown cells was examined using clonogenic cell survival assay. Bars represent the numbers of the colonies (lower panels). (D) The *in vivo* glioma cell growth of U87 cell line was determined by xenograft model assay, NMI overexpression (left panels) and knockdown (right panels) cells were transplanted into nude mice, and monitored every 2-3 days. Tumor growth curves were determined (upper panels), tumor weight (center panels) and size (lower panels) were shown. Each experiment was performed in triplicate. Error bars represent the SEM of the mean value. Statistical analysis was determined by two tailed student's t-test. *, *P*<0.05; **, *P*<0.01; ***, *P*<0.001; #, *P*<0.0001.

To explore the biological significance of NMI in glioma, we investigated whether NMI could affect cell proliferation. First, NMI stably overexpressed or silenced U251 and U87 cells were established by lentiviruses infection, while the empty vector (vector) or shRNA targeting LacZ (shLacZ) served as control groups respectively. The efficiency of NMI overexpression and knockdown was validated by real-time PCR (Supplementary Figure S2) and western blot (Figure 4A) analyses.

We then studied the impact of NMI on glioma cell proliferation *in vitro*. Cell growth was first determined by CCK-8 assay within a 5-day period monitoring. The results showed that enforced expression of NMI significantly promoted the proliferation of U251 and U87 cells compared with control groups (Figure 4B, upper panels) whereas the blockade of endogenous NMI expression in both U251 and U87 cells markedly inhibited cell growth in comparison with the controls (Figure 4B, lower panels). In addition, we further examined the effect of ectopic NMI expression on long-term cell growth using a clonogenic assay in U251 cells. The colony forming ability of U251 cells with overexpressed NMI was evidently enhanced (Figure 4C, left panels), whereas colonies number of NMI silenced U251 cells dramatically decreased compared to control cells (Figure 4C, right panels). The effects of NMI overexpression and deletion on glioma cells were also validated in another cell line, namely A172 (Supplementary Figure S3). These results suggest that NMI promotes the proliferation of glioma cells *in vitro,* depletion of which diminishes glioma cell growth.

### Ectopic NMI expression affects glioma cell growth *in vivo*

We further examined the role of NMI in glioma growth *in vivo* using a subcutaneous xenograft mouse model. Stable NMI expressed or NMI silenced U87 cells and their corresponding control cells were subcutaneously injected into right flanks of immunodeficient nude mice. Similar to our former findings, overexpression of NMI enhanced tumor growth during the whole period (Figure 4D, upper left panel). Tumors of the NMI overexpression group were also substantially heavier and larger than those of the control group (Figure 4D, center left and lower left panels). In contrast, abrogation of NMI considerably retarded the xenograft tumor growth and resulted in apparently lighter and smaller tumors than those of the control group (Figure 4D, right panels). Taken together, these findings reveal an important role of NMI in regulation of glioma growth *in vivo*.

### NMI regulates G1/S phase progression of glioma cell cycle

**Figure 5:**
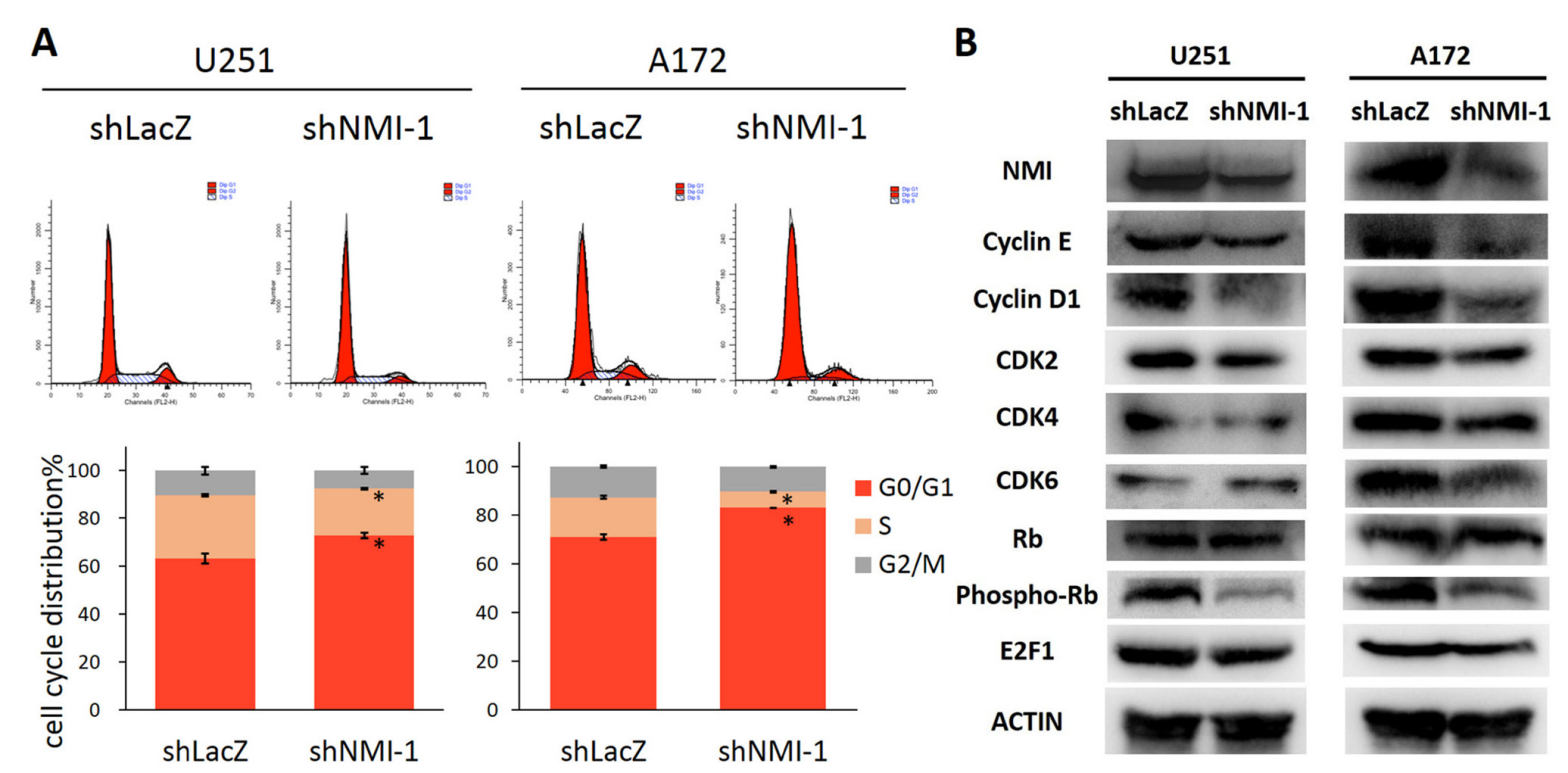
NMI regulates G1/S phase progression of glioma cell cycle (A) The cell cycle distribution of U251 (left panels) and A172 (right panels) glioma cells after depleting endogenous NMI expression. Error bars represent the SEM of the mean value. Statistical analysis was determined by two tailed student's t-test. *, *P*<0.05. (B) The effects of NMI depletion on cell cycle related proteins in the G1/S transition were determined by western blot analysis. ACTIN served as a loading control.

To elucidate the mechanism by which NMI influences glioma cell proliferation, we next determined whether cell cycle distribution was regulated. The effects of NMI depletion on cell cycle progression of U251 and A172 glioma cells were characterized by flow cytometric analyses. Compared with the control shRNA infected cells, NMI silenced glioma cells showed a substantial increase in the percentage of the G0/G1 phase and marked decrease in the S phase (Figure 5A). To investigate the underlying molecular events in changes of cell cycle, we further measured expression levels of several important proteins involved in cell cycle by western blot assays. As a result, depletion of NMI triggered reduction of Cyclin E, Cyclin D1, CDK2, CDK4, CDK6 and E2F1 proteins, and dephosphorylation of Rb (Figure 5B). Together, these results suggest that knockdown of endogenous NMI could induce G0/G1 cell cycle arrest in glioma cells.

### Interaction and mutual regulation of NMI and STAT1

**Figure 6:**
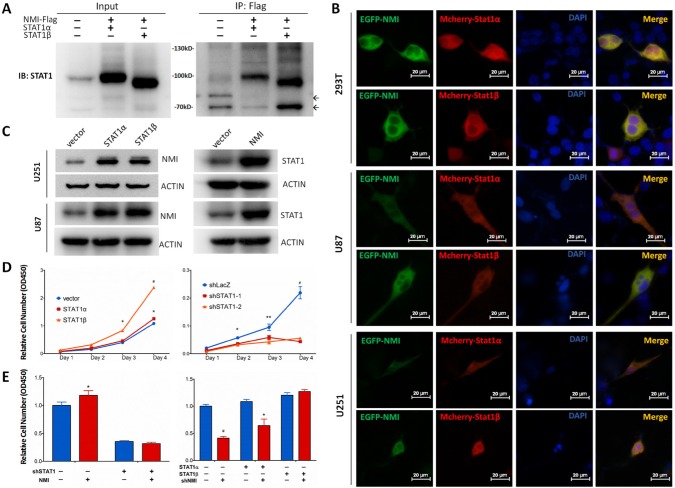
NMI interacts with STAT1 and promotes glioma cell proliferation via regulating STAT1 expression (A) Interaction between NMI and STAT1 was validated by co-immunoprecipitation assay. Input or Flag-beads immunoprecipitated fraction of lysate of U251 cells infected with NMI-Flag or STAT1 expressing lentivirus were immunobloted with STAT1 antibody. Molecular weight marker is as indicated. Non-specific bands are indicated by arrows. (B) Colocalization of NMI-GFP (green) and STAT1-mCherry (red) was confirmed in 293T, U251 and U87 cell lines. Cells were transfected with corresponding plasmids and observed with a fluorescence microscopy 48 hours later. Scale bars: 20 μm. (C) Overexpression of NMI or STAT1 increased the protein expression level of the other one in U251 and U87 cell lines. Cells were lysed and subjected to Western blot analysis. ACTIN served as a loading control. (D) Overexpression (left panel) or knockdown (right panel) of STAT1 promoted or inhibited U251 cell proliferation respectively, examined by CCK-8 assay. (E) Promotion of human glioma cells proliferation mediated by NMI is dependent on its regulation on STAT1. U251 cells infected with STAT1-deletion or control lentivirus were transduced with NMI-expressing or control lentivirus (left panel). STAT1 expressing or control cells were infected with NMI-suppression or control lentivirus (right panel). Cck-8 assay was performed 4 days after seeding on plates to assess cell proliferation. Each experiment was performed in triplicate. Error bars represent the SEM of the mean value. Statistical analysis was determined by two tailed student's t-test. *, *P*<0.05; **, *P*<0.01; ***, *P*<0.001; #, *P*<0.0001.

Although NMI has been shown to interact with STAT1, a member of JAK/STAT pathway [26], none of that was reported in glioma. Moreover, the former view of STAT1, which generally considered it as a tumor suppressor [43], has been shattered by emerging evidences that elevated STAT1 was associated with facilitated tumor progression [44]. Hence, we assessed the relationship between NMI and STAT1 in glioma. Initially, we found that the mRNA expression of NMI and STAT1 was positively correlated with each other in the TCGA cohort (Supplementary Figure S4). Furthermore, to confirm the interaction of NMI and STAT1, a co-IP assay was applied, in which U251 glioma cells were infected with lentiviruses carrying Flag-tagged NMI and STAT1 expressing vectors while empty vector served as a control. As shown in Figure 6A, both STAT1α and STAT1β (two isoforms of the STAT1 protein) could be readily detected in the Flag-NMI immunoprecipitates of cells overexpressing both Flag-NMI and STAT1 but not in control cells. To further determine whether NMI localizes in the same cellular compartment of STAT1, we co-transfected U251, U87 and 293T cells with pEGFP-NMI and pmCherry-STAT1 expression plasmids and examined the locations of NMI and STAT1 under fluorescence microscopy. We found that NMI and STAT1 showed dominant expression in the cytoplasm rather than the nucleus with both of the fluorescent signals merged with each other (Figure 6B), indicating the co-localization of NMI and STAT1 within glioma cells.

We next asked whether NMI and STAT1 could regulate each other. To this end, we overexpressed STAT1 or NMI in U251 and U87 cells, followed by detection of the protein expression of the other one. As shown in Figure 6C, we observed considerable upregulation of NMI in response to STAT1 overexpression. Moreover, NMI overexpression also resulted in remarkable increase of STAT1. Collectively, these data suggest that NMI and STAT1 not merely interact with each other in glioma cells, but have a mutual regulation.

### NMI regulates glioma growth *via* STAT1

To explore the role of STAT1 in glioma growth, we assessed proliferation of U251 cells overexpressed or lost STAT1 by CCK-8 assays. The efficiency of STAT1 silence was validated by real time PCR and western blot assays (Supplementary Figure S5). We found that overexpression of STAT1α increased cell growth slightly, which reached statistical significance at day 4 (*P* < 0.05, Figure 6D, left panel) relative to the empty vector control, while STAT1β enhanced cell proliferation evidently (Figure 6D, left panel). In contrast, depletion of STAT1 expression could dramatically inhibit glioma cell growth in comparison with the control (Figure 6D, right panel).

Given the role of NMI in regulation of glioma cell growth and STAT1 expression as mentioned above, and regulatory role of STAT1 in glioma cell proliferation, we hypothesized that NMI may affect glioma cell growth *via* regulation of STAT1. To test our hypothesis, we evaluated whether STAT1 could alter the effect of NMI on glioma cell growth. As shown in Figure 6E (left panel), by comparing the first two columns, NMI enhanced U251 cell growth significantly as expected. However, the enhancement of cell growth was completely abrogated by additional depletion of STAT1 (comparing the last two columns with the first two, Figure 6E, left panel). Furthermore, as shown in Figure 6E (right panel), the inhibition of cell proliferation mediated by NMI depletion (comparing the first two columns) was also partly attenuated by accompanying overexpression of STAT1α (comparing the middle two groups with the first two), and totally diminished by simultaneous overexpression of STAT1β (comparing the last two groups with the first two). Taken together, these data indicate that the effect of NMI on glioma cell proliferation is dependent on its regulation of STAT1.

## DISCUSSION

Despite recent advances in clinical therapies, the overall outcome of patients with GBM changed little over the past two decades [6]. Thus, new effective prognostic biomarkers and therapeutic targets for GBM are of urgent need. The present research identified NMI as a novel prognostic factor for GBM, which was also a regulator of tumor proliferation.

NMI is a protein identified to interact with a variety of proteins involved in tumor development, progression, inflammation and immune regulation [25, 26, 29, 30, 32, 33]. However, no study has reported the prognostic significance of NMI in human cancers including glioma. Hence, our study aimed to unveil the indispensable roles of NMI in glioma progression. Our TMA analyses revealed evident up-regulation of NMI protein in human glioma tissues, which was significantly increased according to tumor grade. The remarkable augmentation of NMI expression was confirmed on the mRNA level in a subset of this cohort using real-time PCR assays. We also explored the clinical significance of NMI, and found that it may serve as an independent prognostic factor. Due to differences of genetic background between populations [14], we subsequently validated these findings in another two independent cohorts, the Rembrandt [18] and TCGA [19] cohorts, with larger sample sizes. These data revealed that NMI is a significant prognostic factor and might play an important role in glioma progression.

Characterized by particular molecular heterogeneity, glioblastoma has recently been classified into distinct subtypes based on gene expression profiles. In TCGA data analyses, we found a significantly reduced expression of *NMI* in the proneural subtype compared to other subtypes. The reason of the relative lower expression in the proneural subtype (still higher than the normal controls) remains unclear. A glioma-CpG island methylator phenotype (G-CIMP) was identified as a distinct subgroup, which belonged to the proneural subtype and predicted a better prognosis for GBM patients [24]. Thus we asked whether lower expression of *NMI* was correlated to the G-CIMP subgroup. Moreover, G-CIMP was tightly correlated with *IDH1* mutation [24], which was recently identified as an important diagnostic and prognostic molecular biomarker of human glioma [45-50] and believed to be sufficient to establish G-CIMP [51]. So we further analyzed the expression profile of *NMI* according to G-CIMP and *IDH1* mutation, and found significantly lower *NMI* expression in the G-CIMP subgroup and *IDH1* mutated subgroup. However, even in non-G-CIMP patients within the proneural subtype, *NMI* expression was still significantly lower than non-proneural subtypes, indicating that G-CIMP only partially contributed to the lower *NMI* expression in the proneural subtype. Within the G-CIMP subgroup, there was no difference of *NMI* expression between *IDH1* wild-type and mutated patients, suggesting that the observation of lower *NMI* expression in *IDH1* mutated subgroup was indeed due to the effect of G-CIMP. Given that G-CIMP was defined as concerted hypermethylation at a large number of loci, we further addressed whether the expression profile of *NMI* was associated with its methylation level. Consequently, we discovered successively increased methylation level of *NMI* gene in non-proneural subtypes, the proneural non-G-CIMP subgroup and the G-CIMP subgroup, which was completely consistent with their corresponding expression level, suggesting a potential epigenetic mechanism on regulation of *NMI* expression. However, further investigations are underway to examine upstream regulation of *NMI* expression in gliomas.

We also attempted to explore the relationship of *NMI* with common molecular aberrations of GBM. Interestingly, we observed enhanced *NMI* expression in *PTEN* deleted or *EGFR* amplified patients. We further analyzed the impact of *NMI* expression on patients' survival stratified by these molecular features. It was rather remarkable that, *NMI* expression could divide patients with wild-type *PTEN* or *EGFR* into two subsets with completely distinct clinical outcome, which was more pronounced than in all patients. However, *NMI* could not predict prognosis for patients with deleted *PTEN* or amplified *EGFR*, suggesting that the prognostic value of *NMI* was dependent on status of *PTEN* and *EGFR* alteration. In other words, patients with low *NMI* expression and concomitant no genetic aberration of *PTEN* or *EGFR* may have rather favorable clinical outcome, the underlying mechanisms of which warranted further perspective studies.

Current understandings of NMI revealed its dual roles in tumor growth. On one hand, it facilitated tumor proliferation in several cancer cell lines including myeloid leukemias and pancreatic ductal adenocarcinomas [25]. On the other hand, NMI was recently indicated as a potential tumor suppressor in breast cancer [39]. But it is worth noting that the former research was only performed in established cell lines, while the latter study was only based on the overexpression of NMI. In addition, due to the heterogeneity and different genetic background of tumors, it is possible that one gene plays different roles in different temporal and spatial context [52]. Hence, the functions and mechanisms of NMI in tumor progression are still far from elucidation. In our study, we observed that NMI overexpression promoted cell proliferation, whereas NMI depletion dramatically hampered cell growth *in vitro* and *in vivo*. In addition, the effects of NMI on glioma cell growth were possibly mediated by its regulation on G1/S progression of cell cycle, which was further validated by accompanied changes of cell cycle related proteins. These results were consistent with our clinical data that high NMI expressers had shorter survival, which further highlighted the therapeutic potential of NMI in GBM treatment. Moreover, since none of the existing reports took advantage of the clinical samples, our study provided valuable evidences for the functional clarification of NMI in GBM.

Previous finding has shown the interaction between NMI and STAT1 [26], yet none was reported in glioma. Consistently, our study confirmed that NMI interacted with STAT1, and these two proteins co-localized in the cytoplasm, while the mechanisms by which NMI regulated STAT1 needed more investigation. JAK/STAT pathway participates in multiple cellular processes including those related to tumor progression. Of all STATs, STAT1 is traditionally classified as a tumor suppressive protein which is thought to play a key role in immune surveillance of tumors [43]. However, accumulating evidences have correlated upregulated STAT1 with cellular resistance to DNA-damaging agents and increased tumor progression in multiple types of cancers [44, 53, 54]. In addition, the expression of STAT1 was reported to predict poor clinical outcome in GBM [22]. Our study confirmed that STAT1 overexpression evidently enhanced GBM cell proliferation whereas depletion of STAT1 considerably impeded cell growth. Moreover, we also demonstrated that the NMI induced glioma cell proliferation was dependent on STAT1, whereas the differences between the two isoforms of STAT1 were not well understood. Considering that NMI has been reported to interact with various proteins [28-35], it is reasonable to assume that besides STAT1, other interactors of NMI may also be involved in development and progression of human glioma, which warrant further investigation.

In summary, we conclude that NMI is significantly elevated in human GBMs, and predicts shorter overall and progression-free survival for patients with GBM as a novel and independent prognostic factor, as found in TMA analysis of a Chinese cohort and confirmed in two independent international cohorts. Furthermore, the expression profile and clinical significance of *NMI* are associated with several molecular features of GBM. Moreover, NMI plays an important role in glioma cell growth both *in vitro* and *in vivo*, suggesting its potential application as a therapeutic target for glioma.

## METHODS

### Patients and tissue samples

For the Chinese TMA cohort, human glioma tissue specimens of different WHO grades were obtained at the time of surgery at the Department of Neurosurgery in Changzheng Hospital, Second Military Medical University (SMMU), between January 2000 and December 2010. Normal brain tissue samples were taken from trauma patients for whom partial resection of normal brain was required as decompression treatment for their severe head injuries. Tumor histology was confirmed independently by two neuropathologists. All the patients or their legal surrogates were asked for permission with written informed consent to the surgical procedures and the use of resected tissue specimens. The selection criteria of this study were as follows: 1) the subject had no history of other tumors; 2) the subject had complete clinical data, including age, gender, clinical manifestations, extent of resection, histological type and pathological grade; 3) the subject underwent evaluation by enhanced head MRI scans for tumor relapse or progression after surgery at least once every six months; 4) the tissue samples were of sufficient quality for experimental use. The study protocol and acquisition of tissue specimens were approved by the Specialty Committee on Ethics of Biomedical Research, SMMU, Shanghai, China.

### Tissue microarray construction and Immunohistochemistry

Formalin-fixed, paraffin-embedded tissues of 209 specimens with different grades of astrocytic glioma and 16 normal tissue samples were used to construct an tissue microarray (TMA) as described previously [55, 56] (Shanghai Biochip Company). An central independent neuropathological review was performed for tumor diagnosis before TMA construction. The slides of each case were evaluated, and areas corresponding to the most cellular and diagnostic regions were selected after verification with hematoxylin and eosin (H&E) staining. 1.5 mm core punch samples were taken from specimens and cut as 4-μm-thick sections, which were then deparaffinized. Endogenous HRP activity was blocked with 3% H_2_O_2_. Antigen retrieval was achieved by boiling in sodium citrate buffer (pH 6.0). After blocking in 10% normal goat serum, immunostaining was performed using a mouse anti-NMI monoclonal antibody (ab56437; Abcam) at 1:650 dilution. Finally, the visualized signal was developed with 3,3′-diaminobenzidine (DAB) and the slides were counterstained in hematoxylin. The sections incubated with normal mouse serum instead of the primary antibody were used as negative controls.

The results of immunohistochemical staining were evaluated by two independent pathologists in a blinded manner without prior knowledge of the clinical information of the patients. The evaluation of the staining density, intensity and the calculation of total immunoreactivity score were described previously [57]. Briefly, the percentage of positive staining cells was scored 0-3 (0 points for no cells stained, 1 points for < 25%, 2 points for 25-75%, and 3 points for > 75% of cells stained ), and the intensity of immunoreactivity was also graded on a scale of 0-3 (0 for no immunostaining, 1 for light-brown color, 2 for medium-brown color, and 3 for dark-brown color ). The two scores were then multiplied to yield a total immunoreactivity score regarding the expression of NMI protein in a sample. Negative cases (−) had a total score of 0, weakly positive (+) cases had a total score of 1-2, moderately positive (++) cases had a score of 3-4, and strongly positive (+++) cases had a total score of 6-9. NMI expression was denoted as low (−,+) or high (++,+++) to divide the patients into two groups.

### RNA extraction, cDNA synthesis, and quantitative real-time PCR

Fresh-frozen tissues from 42 human glioma patients and 10 normal brain samples were used for total RNA extraction using the Trizol reagent (Invitrogen) according to the manufacturer's instructions. Reverse transcription of total RNA was conducted using ReverTra Ace qPCR RT Master Mix (Toyobo). Quantitative real-time PCR was performed using THUNDERBIRD SYBR qPCR Mix (Toyobo) on ABI PRISM 7900HT instruments (Applied Biosystems). The amplification was done in a total volume of 10 μl with the following steps: an initial denaturation step at 95°C for 5 minutes, followed by 40 cycles of denaturation at 95°C for 15 seconds and elongation at 60°C for 45 seconds. A melting curve analysis of each sample was used to check the specificity of amplification, and each sample was assayed in triplicate. Glyceraldehyde-3-phosphate dehydrogenase (*GAPDH*) was used as the endogenous control, and the 2^−ΔΔCt^ method [58] was used as relative quantification measure of differential expression. All PCR primers were listed in Supplementary Table S5.

### *In silico* analyses of TCGA and Rembrandt data

Another two independent datasets of GBMs, the Repository of Molecular Brain Neoplasia Data (Rembrandt) [18] cohort (N = 371 ) and The Caner Genome Atlas (TCGA) [19] cohort (N = 528 for the Affymetrix platform and N = 396 for the Agilent platform), were also included in the present study. Expression and clinical significance of *NMI* gene were analyzed in the Rembrandt database (http://caintegrator.nci.nih.gov/rembrandt/). For the TCGA cohort, multidimensional data of gene expression, clinical information, common mutations, copy number alterations, methylation levels and molecular subtypes were obtained from TCGA data portal (http://tcga-data.nci.nih.gov/tcga/) and cBioPortal (http://www.cbioportal.org/public-portal/), to analyze the expression profile and prognostic value of *NMI* gene, and their relationships with these molecular features.

### Cell culture and transfection

U251, U87 and A172 human malignant glioma cell lines, and 293T human embryonic kidney cell line were purchased from Cell Bank of Chinese Academy of Sciences (Shanghai, China) where they were authenticated by mycoplasma detection, DNA-Fingerprinting, isozyme detection and cell vitality detection. All these cells were cultured in Dulbecco's modified Eagle's medium (DMEM; Life Technologies) supplemented with 10% fetal bovine serum (FBS; Life Technologies) and penicillin (100 units/ml)/streptomycin (100 μg/ml) (Life Technologies) and maintained at 37°C in an atmosphere of humidified air containing 5% CO_2_.

Transfection procedures were described in our previous study [59]. Briefly, plasmid DNA were introduced into appropriate cells cultured in 24-well plates for 48 hours using Lipofectamine 2000 (Invitrogen) according to the manufacturer's instructions.

### Plasmids and constructions

High fidelity enzyme KOD plus neo (Toyobo) was used for PCR amplification for all the constructions, which were verified by sequencing. For co-localization study, NMI and STAT1 were fused to GFP or mCherry fluorescent protein. In detail, the coding sequence of the *NMI* gene was amplified from cDNA of U87 cell line by PCR and cloned into pEGFP-C3 plasmid (Clontech) at the *Xho*I and *Bam*HI restriction sites at the C-terminal of GFP in frame to generate the plasmid GFP-NMI, whereas the two different transcript variants of the *STAT1* gene were cloned into pmCherry-C1 plasmid (Clontech) at the *Bgl*II and *Kpn*I restriction sites at C-terminal for the expression of mCherry-fused STAT1 isoform α or β designated mCherry-STAT1-V1 or mCherry-STAT1-V2, respectively. GFP-NMI and mCherry-STAT1 (V1 or V2) were co-transfected in different cell lines, and fluorescence images were captured using Nikon ECLIPSE Ni-U microscopy (Nikon, Japan) and managed using NIS Elements D4.00 software (Nikon, Japan).

To overexpress NMI or STAT1, corresponding coding sequences were subcloned from abovementioned GFP-NMI or mCherry-STAT1 constructs into a lentiviral vector pCDH-CMV-EF1-copGFP (pCDH; System Biosciences) at the *Xba*I – *Bam*HI and *Nhe*I – *Bam*HI restriction sites respectively, to generate constructions named pCDH-NMI, pCDH-STAT1-V1 and pCDH-STAT1-V2. The pCDH-Flag-NMI construction was generated by fusing a sequence encoding a Flag tag at the N-terminal of NMI encoding sequence.

To interfere NMI or STAT1 expression, target sequences were selected from the Public TRC Portal (http://www.broadinstitute.org/rnai/public/) [60]. The 21-nucleotide target sequences of NMI and STAT1 were as follows: shNMI-1(clone ID: TRCN0000022050): 5′-GCCAAGCCAGTTCCATTAAAT-3′; shNMI-2(clone ID TRCN0000293456): 5′-TTAACCCGGATTACTGTAAAT-3′ ; shSTAT1-1(Clone ID TRCN0000004265): 5′-CCCTGAAGTATCTGTATCCAA-3′ ; shSTAT1-2(Clone ID TRCN0000004267): 5′-CTGGAAGATTTACAAGATGAA-3′. Sequence against the LacZ gene served as a control designated shLacZ: 5′-GGATCAGTCGCTGATTAAA-3′ [61]. Corresponding sense and anti-sense oligonucleotides (Supplemental Table S5) were synthesized, annealed and cloned into the *Hpa*I - *Xho*I sites of pLL3.7 lentiviral vector [62].

### Lentivirus production and transduction

Lentiviral production and transduction were conducted as previously described [63]. Briefly, 293T cells were co-transfected with the lentiviral expression vector pCDH (empty vector as a control) and corresponding constructions together with lentiviral packaging plasmids pLP/VSVG, pLP1 and pLP2 for overexpression of target genes, and with lentiviral vectors pLL3.7-shRNAs and corresponding packaging vectors psPAX2 and pMD2.G for gene silence. The supernatants of lentiviral particles were collected 48 hours post transfection, filtered through 0.45-μm syringe filters (Millipore) to remove cell debris and used either immediately or stored at −80°C. U251 and U87 human glioma cells were plated in 24-well plates overnight to achieve 30% confluence the following day, and infected in the presence of 4 μg/mL polybrene (Sigma-Aldrich) with the lentiviruses carrying the pCDH expression vector or shRNAs against NMI or STAT1 along with their corresponding controls. Overexpression and knockdown of target genes were validated by real-time PCR and Western blot analysis.

### Cell proliferation assay and Clonogenic assay

For cell proliferation assay described previously [64], different cell lines were seeded in 96-well plates (about 2000 cells/well) in sextuple. Cells were allowed to grow for 4 to 5 days and cell proliferation analysis was performed by Cell Counting Kit-8 (CCK-8; Dojindo Laboratories) assay at different time points according to the manufacturer's instructions. After an incubation of 2 hours at 37°C, absorbance was measured at 450 nm using a microplate reader iMark (Bio-Rad). The absorbance at 630 nm was used as a reference.

The effects of NMI overexpression or knockdown on long-term growth of glioma cells were assessed by clonogenic assays as preciously described [65]. Different cell lines were seeded in 6-well plates (about 2000 cells/well) in triplicate. The medium was replaced every 3 days, and cells were allowed to grow for 2 weeks before being fixed with ice-cold methanol and stained with Giemsa's stain, and the number of colonies (defined as cell clusters consisting of at least 50 cells) was then counted.

### Cell cycle analysis

Cell cycle distribution was analyzed as described previously [64]. Briefly, glioma cells were infected by lentiviruses delivering shRNAs against NMI or LacZ as a negative control, and harvested 4 days later, followed by propidium iodide (10μg/mL) staining in the presence of RNase (10μg/mL) for 30 minutes at 4°C in the dark. The fractions of viable cells in G0/G1, S and G2 phases of cell cycle were determined using a FACs flow cytometer and Cell Quest FACS system (Becton-Dickinson).

### Subcutaneous xenograft model

Subcutanious xenograft model of malignant glioma was established as previously described [66-71]. Briefly, approximately 5×10^6^ of U87 cells infected with NMI overexpression or deletion lentiviruses were injected subcutaneously into the right flanks of 4-week-old male athymic nude mice (BALB/c nu/nu; Slac Laboratory Animal). For each group 6 mice were used. Tumor size was monitored 3 times a week using a vernier caliper and determined by measuring the length (*l*) and the width (*w*), followed by calculating the volume (*V* = *lw*^2^/2). Mice were sacrificed by dislocation of vertebrae when the longest diameter of the largest xenograft of a group reached 3 cm, and tumor weights were determined. All animal studies were carried out in accordance with the protocols approved by the Institutional Animal Care and Use Committee of Fudan University.

### Co-immunoprecipitation and Western blot

Co-immunoprecipitation (co-IP) was conducted as previously described [72]. Briefly, U251 cells stably overexpressing NMI-Flag and STAT1 (α or β) via lentivirus infection were lysed in NP-40 lysis buffer [50 mM Tris-HCl pH7.5, 150 mM NaCl, 1% (v/v) NP-40] with protease inhibitors cocktail (Sigma-Aldrich) added freshly. Immunoprecipitation of NMI-Flag was performed by adding 10 μl of anti-Flag M2 magnetic beads (Sigma-Aldrich) per 0.6 to 0.8 mg of protein lysate. Cell extracts with anti-Flag M2 magnetic beads were incubated overnight at 4°C on a rocking platform. Finally the immunocomplex was washed four times using the lysis buffer and boiled in the SDS loading buffer followed by western blot analysis.

Western blot was performed as described previously [73]. Briefly, cells were lysed in the radioimmunoprecipitation assay (RIPA) buffer [50 mM Tris-HCl pH 8.0, 150 mM NaCl, 1% (v/v) NP-40, 0.5% (w/v) Sodium deoxycholate, 0.1% (w/v) SDS] with protease inhibitors cocktail (Sigma) added freshly. The lysates were separated by 10% SDS-PAGE and transferred to polyvinylidene difluoride membranes (Millipore), which were blocked in 5% milk for 1 hour and then probed with antibodies against NMI(1:200; ab56437; Abcam), STAT1 (1:1000; #9172; Cell Signaling Technology), Cyclin E (1:500; PC438-500UG; Oncogene), Cyclin D1 (1:1000; #2926; Cell Signaling Technology), CDK2 (1:1000; 60312-1-lg; Proteintech), CDK4 (1:1000; 11026-1-AP; Proteintech), CDK6 (1:2000; 19117-1-AP; Proteintech), Rb (1:1000; 17218-1-AP; Proteintech), phospho-Rb Ser807/811 (1:1000; #8516; Cell Signaling Technology), E2F1 (1:1000; 3240-1; Epitomics), or ACTIN (1:4000; M20010; Abmart) as a loading control. Blots were developed with Immobilon Western Chemiluminescent HRP Substrate (Millipore) and visualized on G: Box Chemi XR5 (Syngene).

### Statistical analysis

Differences between two groups were analyzed by two-tailed student's t-test. For survival analysis, overall survival (OS) was defined as the elapsed time between diagnosis and death or the last follow-up (if death was not observed during the follow-up period), and progression-free survival (PFS) was defined as the time from diagnosis to the date of tumor recurrence or further growth of residual tumor detected by enhanced MRI scan, or the date of death or the last contact of the patient (if progression didn't happen). Survival curves were estimated by the Kaplan-Meier method and compared using the log-rank test. To construct a model for the prediction of survival, univariate and multivariate Cox proportional-hazards regression analyses were performed, in which clinical variables with *P* < 0.05 in univariate analysis were pooled into multivariate analysis. For the TCGA cohort, receiver operating characteristic (ROC) curve was used to determine the cutoff point of *NMI* expression levels in the survival analyses, and the score localized closest to the point at both maximum sensitivity and specificity was selected [74]. Correlations between continuous variables were assessed by linear regression model, and Pearson r and *P* value were calculated. SPSS 15.0 software (SPSS Inc.) was used for all statistical analysis and *P* < 0.05 was considered statistically significant for all tests. Figures were plotted in SPSS 15.0 or GraphPad Prism 5.

## SUPPLEMENTARY MATERIAL FIGURES AND TABLES



## References

[1] Ostrom QT, Gittleman H, Farah P, Ondracek A, Chen Y, Wolinsky Y, Stroup NE, Kruchko C, Barnholtz-Sloan JS (2013). CBTRUS statistical report: Primary brain and central nervous system tumors diagnosed in the United States in 2006-2010. Neuro Oncol.

[2] Westphal M, Lamszus K (2011). The neurobiology of gliomas: from cell biology to the development of therapeutic approaches. Nat Rev Neurosci.

[3] Gilbert MR, Dignam JJ, Armstrong TS, Wefel JS, Blumenthal DT, Vogelbaum MA, Colman H, Chakravarti A, Pugh S, Won M, Jeraj R, Brown PD, Jaeckle KA, Schiff D, Stieber VW, Brachman DG (2014). A randomized trial of bevacizumab for newly diagnosed glioblastoma. N Engl J Med.

[4] Ohgaki H, Kleihues P (2005). Epidemiology and etiology of gliomas. Acta Neuropathol.

[5] Stupp R, Mason WP, van den Bent MJ, Weller M, Fisher B, Taphoorn MJ, Belanger K, Brandes AA, Marosi C, Bogdahn U, Curschmann J, Janzer RC, Ludwin SK, Gorlia T, Allgeier A, Lacombe D (2005). Radiotherapy plus concomitant and adjuvant temozolomide for glioblastoma. N Engl J Med.

[6] Tanaka S, Louis DN, Curry WT, Batchelor TT, Dietrich J (2013). Diagnostic and therapeutic avenues for glioblastoma: no longer a dead end?. Nat Rev Clin Oncol.

[7] Van Meir EG, Hadjipanayis CG, Norden AD, Shu HK, Wen PY, Olson JJ (2010). Exciting new advances in neuro-oncology: the avenue to a cure for malignant glioma. CA Cancer J Clin.

[8] Babae N, Bourajjaj M, Liu Y, Van Beijnum JR, Cerisoli F, Scaria PV, Verheul M, Van Berkel MP, Pieters EH, Van Haastert RJ, Yousefi A, Mastrobattista E, Storm G, Berezikov E, Cuppen E, Woodle M (2014). Systemic miRNA-7 delivery inhibits tumor angiogenesis and growth in murine xenograft glioblastoma. Oncotarget.

[9] Chen L, Liu X, Zhang HY, Du W, Qin Z, Yao Y, Mao Y, Zhou L (2014). Upregulation of chemokine receptor CCR10 is essential for glioma proliferation, invasion and patient survival. Oncotarget.

[10] Kim JW, Kim JY, Kim JE, Kim SK, Chung HT, Park CK (2014). HOXA10 is associated with temozolomide resistance through regulation of the homologous recombinant DNA repair pathway in glioblastoma cell lines. Genes Cancer.

[11] Paul I, Bhattacharya S, Chatterjee A, Ghosh MK (2013). Current Understanding on EGFR and Wnt/beta-Catenin Signaling in Glioma and Their Possible Crosstalk. Genes Cancer.

[12] Kitanaka C, Sato A, Okada M (2013). JNK Signaling in the Control of the Tumor-Initiating Capacity Associated with Cancer Stem Cells. Genes Cancer.

[13] Sun Y, Zhang W, Chen D, Lv Y, Zheng J, Lilljebjorn H, Ran L, Bao Z, Soneson C, Sjogren HO, Salford LG, Ji J, French PJ, Fioretos T, Jiang T, Fan X (2014). A glioma classification scheme based on coexpression modules of EGFR and PDGFRA. Proc Natl Acad Sci U S A.

[14] Yan W, Zhang W, You G, Zhang J, Han L, Bao Z, Wang Y, Liu Y, Jiang C, Kang C, You Y, Jiang T (2012). Molecular classification of gliomas based on whole genome gene expression: a systematic report of 225 samples from the Chinese Glioma Cooperative Group. Neuro Oncol.

[15] Zhang JX, Han L, Bao ZS, Wang YY, Chen LY, Yan W, Yu SZ, Pu PY, Liu N, You YP, Jiang T, Kang CS (2013). HOTAIR, a cell cycle-associated long noncoding RNA and a strong predictor of survival, is preferentially expressed in classical and mesenchymal glioma. Neuro Oncol.

[16] Meng J, Li P, Zhang Q, Yang Z, Fu S (2014). A radiosensitivity gene signature in predicting glioma prognostic via EMT pathway. Oncotarget.

[17] Shi Y, Chen C, Zhang X, Liu Q, Xu JL, Zhang HR, Yao XH, Jiang T, He ZC, Ren Y, Cui W, Xu C, Liu L, Cui YH, Yu SZ, Ping YF (2014). Primate-Specific miR-663 Functions as a Tumor Suppressor by Targeting PIK3CD and Predicts the Prognosis of Human Glioblastoma. Clin Cancer Res.

[18] Madhavan S, Zenklusen JC, Kotliarov Y, Sahni H, Fine HA, Buetow K (2009). Rembrandt: helping personalized medicine become a reality through integrative translational research. Mol Cancer Res.

[19] The Cancer Genome Atlas (TCGA) Research Network (2008). Comprehensive genomic characterization defines human glioblastoma genes and core pathways. Nature.

[20] Rutledge WC, Kong J, Gao J, Gutman DA, Cooper LA, Appin C, Park Y, Scarpace L, Mikkelsen T, Cohen ML, Aldape KD, McLendon RE, Lehman NL, Miller CR, Schniederjan MJ, Brennan CW (2013). Tumor-infiltrating lymphocytes in glioblastoma are associated with specific genomic alterations and related to transcriptional class. Clin Cancer Res.

[21] Lin Y, Chen Y, Wang Y, Yang J, Zhu VF, Liu Y, Cui X, Chen L, Yan W, Jiang T, Hergenroeder GW, Fletcher SA, Levine JM, Kim DH, Tandon N, Zhu JJ (2013). ZIP4 is a novel molecular marker for glioma. Neuro Oncol.

[22] Duarte CW, Willey CD, Zhi D, Cui X, Harris JJ, Vaughan LK, Mehta T, McCubrey RO, Khodarev NN, Weichselbaum RR, Gillespie GY (2012). Expression signature of IFN/STAT1 signaling genes predicts poor survival outcome in glioblastoma multiforme in a subtype-specific manner. PLoS One.

[23] Verhaak RG, Hoadley KA, Purdom E, Wang V, Qi Y, Wilkerson MD, Miller CR, Ding L, Golub T, Mesirov JP, Alexe G, Lawrence M, O'Kelly M, Tamayo P, Weir BA, Gabriel S (2010). Integrated genomic analysis identifies clinically relevant subtypes of glioblastoma characterized by abnormalities in PDGFRA, IDH1, EGFR, and NF1. Cancer Cell.

[24] Noushmehr H, Weisenberger DJ, Diefes K, Phillips HS, Pujara K, Berman BP, Pan F, Pelloski CE, Sulman EP, Bhat KP, Verhaak RG, Hoadley KA, Hayes DN, Perou CM, Schmidt HK, Ding L (2010). Identification of a CpG island methylator phenotype that defines a distinct subgroup of glioma. Cancer Cell.

[25] Bao J, Zervos AS (1996). Isolation and characterization of Nmi, a novel partner of Myc proteins. Oncogene.

[26] Zhu M, John S, Berg M, Leonard WJ (1999). Functional association of Nmi with Stat5 and Stat1 in IL-2- and IFNgamma-mediated signaling. Cell.

[27] Stark GR, Darnell JE (2012). The JAK-STAT pathway at twenty. Immunity.

[28] Zhou X, Liao J, Meyerdierks A, Feng L, Naumovski L, Bottger EC, Omary MB (2000). Interferon-alpha induces nmi-IFP35 heterodimeric complex formation that is affected by the phosphorylation of IFP35. J Biol Chem.

[29] Li H, Lee TH, Avraham H (2002). A novel tricomplex of BRCA1, Nmi, and c-Myc inhibits c-Myc-induced human telomerase reverse transcriptase gene (hTERT) promoter activity in breast cancer. J Biol Chem.

[30] Schlierf B, Lang S, Kosian T, Werner T, Wegner M (2005). The high-mobility group transcription factor Sox10 interacts with the N-myc-interacting protein Nmi. J Mol Biol.

[31] Zhang K, Zheng G, Yang YC (2007). Stability of Nmi protein is controlled by its association with Tip60. Mol Cell Biochem.

[32] Zhang L, Tang Y, Tie Y, Tian C, Wang J, Dong Y, Sun Z, He F (2007). The PH domain containing protein CKIP-1 binds to IFP35 and Nmi and is involved in cytokine signaling. Cell Signal.

[33] Li Z, Hou J, Sun L, Wen T, Wang L, Zhao X, Xie Q, Zhang SQ (2012). NMI mediates transcription-independent ARF regulation in response to cellular stresses. Mol Biol Cell.

[34] Wang J, Wang Y, Liu J, Ding L, Zhang Q, Li X, Cao H, Tang J, Zheng SJ (2012). A critical role of N-myc and STAT interactor (Nmi) in foot-and-mouth disease virus (FMDV) 2C-induced apoptosis. Virus Res.

[35] Wang J, Yang B, Hu Y, Zheng Y, Zhou H, Wang Y, Ma Y, Mao K, Yang L, Lin G, Ji Y, Wu X, Sun B (2013). Negative regulation of Nmi on virus-triggered type I IFN production by targeting IRF7. J Immunol.

[36] Brozzi F, Gerlo S, Grieco FA, Nardelli TR, Lievens S, Gysemans C, Marselli L, Marchetti P, Mathieu C, Tavernier J, Eizirik DL (2014). A Combined “Omics” Approach Identifies N-Myc Interactor as a Novel Cytokine-induced Regulator of IRE1alpha Protein and c-Jun N-terminal Kinase in Pancreatic Beta Cells. J Biol Chem.

[37] Saito Y, Yukawa A, Matozaki M, Mikami H, Yamagami T, Yamagishi N, Kuga T, Hatayama T, Nakayama Y (2014). Nmi interacts with Hsp105beta and enhances the Hsp105beta-mediated Hsp70 expression. Exp Cell Res.

[38] Lebrun SJ, Shpall RL, Naumovski L (1998). Interferon-induced upregulation and cytoplasmic localization of Myc-interacting protein Nmi. J Interferon Cytokine Res.

[39] Fillmore RA, Mitra A, Xi Y, Ju J, Scammell J, Shevde LA, Samant RS (2009). Nmi (N-Myc interactor) inhibits Wnt/beta-catenin signaling and retards tumor growth. Int J Cancer.

[40] Devine DJ, Rostas JW, Metge BJ, Das S, Mulekar MS, Tucker JA, Grizzle WE, Buchsbaum DJ, Shevde LA, Samant RS (2014). Loss of N-Myc interactor promotes epithelial-mesenchymal transition by activation of TGF-beta/SMAD signaling. Oncogene.

[41] Rostas JW, Pruitt HC, Metge BJ, Mitra A, Bailey SK, Bae S, Singh KP, Devine DJ, Dyess DL, Richards WO, Tucker JA, Shevde LA, Samant RS (2014). microRNA-29 negatively regulates EMT regulator N-myc interactor in breast cancer. Mol Cancer.

[42] Brennan CW, Verhaak RG, McKenna A, Campos B, Noushmehr H, Salama SR, Zheng S, Chakravarty D, Sanborn JZ, Berman SH, Beroukhim R, Bernard B, Wu CJ, Genovese G, Shmulevich I, Barnholtz-Sloan J (2013). The somatic genomic landscape of glioblastoma. Cell.

[43] Yu H, Pardoll D, Jove R (2009). STATs in cancer inflammation and immunity: a leading role for STAT3. Nat Rev Cancer.

[44] Hix LM, Karavitis J, Khan MW, Shi YH, Khazaie K, Zhang M (2013). Tumor STAT1 transcription factor activity enhances breast tumor growth and immune suppression mediated by myeloid-derived suppressor cells. J Biol Chem.

[45] Balss J, Meyer J, Mueller W, Korshunov A, Hartmann C, von Deimling A (2008). Analysis of the IDH1 codon 132 mutation in brain tumors. Acta Neuropathol.

[46] Dang L, White DW, Gross S, Bennett BD, Bittinger MA, Driggers EM, Fantin VR, Jang HG, Jin S, Keenan MC, Marks KM, Prins RM, Ward PS, Yen KE, Liau LM, Rabinowitz JD (2009). Cancer-associated IDH1 mutations produce 2-hydroxyglutarate. Nature.

[47] Hartmann C, Meyer J, Balss J, Capper D, Mueller W, Christians A, Felsberg J, Wolter M, Mawrin C, Wick W, Weller M, Herold-Mende C, Unterberg A, Jeuken JW, Wesseling P, Reifenberger G (2009). Type and frequency of IDH1 and IDH2 mutations are related to astrocytic and oligodendroglial differentiation and age: a study of 1,010 diffuse gliomas. Acta Neuropathol.

[48] Nobusawa S, Watanabe T, Kleihues P, Ohgaki H (2009). IDH1 mutations as molecular signature and predictive factor of secondary glioblastomas. Clin Cancer Res.

[49] Yan H, Parsons DW, Jin G, McLendon R, Rasheed BA, Yuan W, Kos I, Batinic-Haberle I, Jones S, Riggins GJ, Friedman H, Friedman A, Reardon D, Herndon J, Kinzler KW, Velculescu VE (2009). IDH1 and IDH2 mutations in gliomas. N Engl J Med.

[50] Zhao S, Lin Y, Xu W, Jiang W, Zha Z, Wang P, Yu W, Li Z, Gong L, Peng Y, Ding J, Lei Q, Guan KL, Xiong Y (2009). Glioma-derived mutations in IDH1 dominantly inhibit IDH1 catalytic activity and induce HIF-1alpha. Science.

[51] Turcan S, Rohle D, Goenka A, Walsh LA, Fang F, Yilmaz E, Campos C, Fabius AW, Lu C, Ward PS, Thompson CB, Kaufman A, Guryanova O, Levine R, Heguy A, Viale A (2012). IDH1 mutation is sufficient to establish the glioma hypermethylator phenotype. Nature.

[52] de la Iglesia N, Konopka G, Puram SV, Chan JA, Bachoo RM, You MJ, Levy DE, Depinho RA, Bonni A (2008). Identification of a PTEN-regulated STAT3 brain tumor suppressor pathway. Genes Dev.

[53] Khodarev NN, Roizman B, Weichselbaum RR (2012). Molecular pathways: interferon/stat1 pathway: role in the tumor resistance to genotoxic stress and aggressive growth. Clin Cancer Res.

[54] Kovacic B, Stoiber D, Moriggl R, Weisz E, Ott RG, Kreibich R, Levy DE, Beug H, Freissmuth M, Sexl V (2006). STAT1 acts as a tumor promoter for leukemia development. Cancer Cell.

[55] Fan S, Meng D, Xu T, Chen Y, Wang J, Li X, Chen H, Lu D, Chen J, Lan Q (2013). Overexpression of SLC7A7 predicts poor progression-free and overall survival in patients with glioblastoma. Med Oncol.

[56] Kononen J, Bubendorf L, Kallioniemi A, Barlund M, Schraml P, Leighton S, Torhorst J, Mihatsch MJ, Sauter G, Kallioniemi OP (1998). Tissue microarrays for high-throughput molecular profiling of tumor specimens. Nat Med.

[57] Ohuchida K, Mizumoto K, Ishikawa N, Fujii K, Konomi H, Nagai E, Yamaguchi K, Tsuneyoshi M, Tanaka M (2005). The role of S100A6 in pancreatic cancer development and its clinical implication as a diagnostic marker and therapeutic target. Clin Cancer Res.

[58] Livak KJ, Schmittgen TD (2001). Analysis of relative gene expression data using real-time quantitative PCR and the 2(−Delta Delta C(T)) Method. Methods.

[59] Zhang S, Chen H, Zhao X, Cao J, Tong J, Lu J, Wu W, Shen H, Wei Q, Lu D (2013). REV3L 3′UTR 460 T>C polymorphism in microRNA target sites contributes to lung cancer susceptibility. Oncogene.

[60] Root DE, Hacohen N, Hahn WC, Lander ES, Sabatini DM (2006). Genome-scale loss-of-function screening with a lentiviral RNAi library. Nat Methods.

[61] Tan Y, Cheung M, Pei J, Menges CW, Godwin AK, Testa JR (2010). Upregulation of DLX5 promotes ovarian cancer cell proliferation by enhancing IRS-2-AKT signaling. Cancer Res.

[62] Rubinson DA, Dillon CP, Kwiatkowski AV, Sievers C, Yang L, Kopinja J, Rooney DL, Zhang M, Ihrig MM, McManus MT, Gertler FB, Scott ML, Van Parijs L (2003). A lentivirus-based system to functionally silence genes in primary mammalian cells, stem cells and transgenic mice by RNA interference. Nat Genet.

[63] Lois C, Hong EJ, Pease S, Brown EJ, Baltimore D (2002). Germline transmission and tissue-specific expression of transgenes delivered by lentiviral vectors. Science.

[64] Li X, Wan X, Chen H, Yang S, Liu Y, Mo W, Meng D, Du W, Huang Y, Wu H, Wang J, Li T, Li Y (2014). Identification of miR-133b and RB1CC1 as Independent Predictors for Biochemical Recurrence and Potential Therapeutic Targets for Prostate Cancer. Clin Cancer Res.

[65] Tang L, Tan YX, Jiang BG, Pan YF, Li SX, Yang GZ, Wang M, Wang Q, Zhang J, Zhou WP, Dong LW, Wang HY (2013). The prognostic significance and therapeutic potential of hedgehog signaling in intrahepatic cholangiocellular carcinoma. Clin Cancer Res.

[66] Brunckhorst MK, Wang H, Lu R, Yu Q (2010). Angiopoietin-4 promotes glioblastoma progression by enhancing tumor cell viability and angiogenesis. Cancer Res.

[67] Djaafri I, Khayati F, Menashi S, Tost J, Podgorniak MP, Sadoux A, Daunay A, Teixeira L, Soulier J, Idbaih A, Setterblad N, Fauvel F, Calvo F, Janin A, Lebbe C, Mourah S (2014). A novel tumor suppressor function of Kindlin-3 in solid cancer. Oncotarget.

[68] Jiang L, Yang YD, Fu L, Xu W, Liu D, Liang Q, Zhang X, Xu L, Guan XY, Wu B, Sung JJ, Yu J (2014). CLDN3 inhibits cancer aggressiveness via Wnt-EMT signaling and is a potential prognostic biomarker for hepatocellular carcinoma. Oncotarget.

[69] Park EY, Chang E, Lee EJ, Lee HW, Kang HG, Chun KH, Woo YM, Kong HK, Ko JY, Suzuki H, Song E, Park JH (2014). Targeting of miR34a-NOTCH1 Axis Reduced Breast Cancer Stemness and Chemoresistance. Cancer Res.

[70] Sainz B, Martin B, Tatari M, Heeschen C, Guerra S (2014). ISG15 Is a Critical Microenvironmental Factor for Pancreatic Cancer Stem Cells. Cancer Res.

[71] Yu H, Ye W, Wu J, Meng X, Liu RY, Ying X, Zhou Y, Wang H, Pan C, Huang W (2014). Overexpression of sirt7 exhibits oncogenic property and serves as a prognostic factor in colorectal cancer. Clin Cancer Res.

[72] Gulati P, Cheung MK, Antrobus R, Church CD, Harding HP, Tung YC, Rimmington D, Ma M, Ron D, Lehner PJ, Ashcroft FM, Cox RD, Coll AP, O'Rahilly S, Yeo GS (2013). Role for the obesity-related FTO gene in the cellular sensing of amino acids. Proc Natl Acad Sci U S A.

[73] Mo W, Zhang J, Li X, Meng D, Gao Y, Yang S, Wan X, Zhou C, Guo F, Huang Y, Amente S, Avvedimento EV, Xie Y, Li Y (2013). Identification of novel AR-targeted microRNAs mediating androgen signalling through critical pathways to regulate cell viability in prostate cancer. PLoS One.

[74] Wang J, Huang Y, Guan Z, Zhang JL, Su HK, Zhang W, Yue CF, Yan M, Guan S, Liu QQ (2014). E3-ligase Skp2 predicts poor prognosis and maintains cancer stem cell pool in nasopharyngeal carcinoma. Oncotarget.

