# Supplementary material for: High expression of N-myc (and STAT) interactor predicts poor prognosis and promotes tumor growth in human glioblastoma
Source: Oncotarget. 2014 Dec 30;6(7):4901–19. doi: 10.18632/oncotarget.3208 (PMC4467123; doi:10.18632/oncotarget.3208)
Supplement: Supplementary file 1 [file oncotarget-06-4901-s001.pdf]

High expression of N-myc (and STAT) interactor predicts poor prognosis and promotes tumor growth in human glioblastoma

Supplementary Material

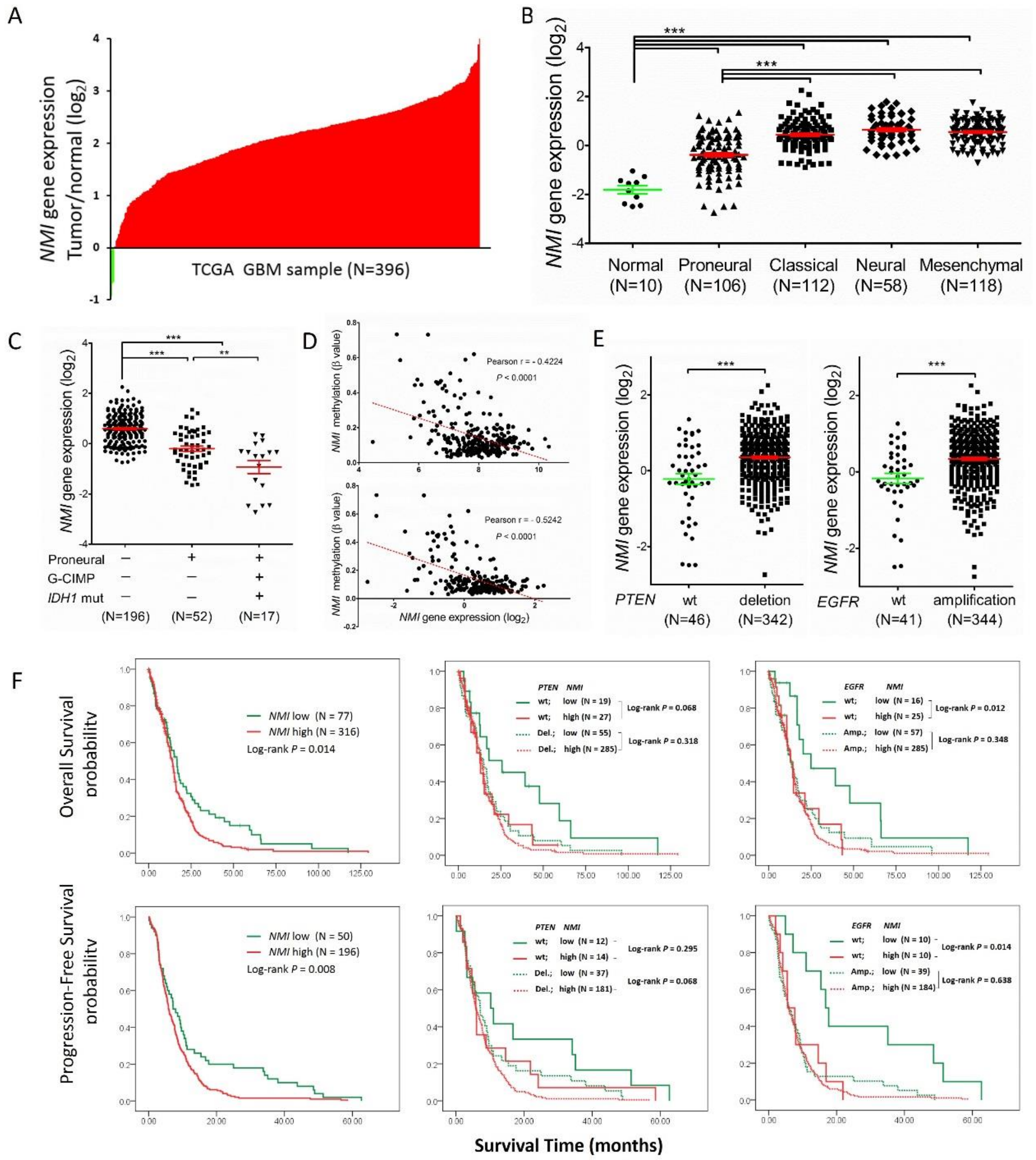

Supplementary Figure S1: *NMI* expression and prognostic significance in GBMs of the TCGA cohort. (A) *NMI* mRNA expression levels were detected in 396 GBM specimens and 10 cases of normal control tissue obtained by TCGA (the Agilent platform). The value represents log2 of gene expression level of each GBM sample to the average mRNA of 10 normal samples. The red samples (>0) indicate that the

mRNA levels of these GBM tissues were higher than the average of normal brain tissues while the green bars (<0) represent GBM sample with lower *NMI* mRNA expression compared to normal tissues. (B) *NMI* mRNA expression levels were compared between normal samples and different molecular subtypes of GBMs as indicated (the Agilent platform). (C) *NMI* expression was compared according to subtype (proneural or not), Glioma-CpG Island Methylation Phenotype (G-CIMP) and *IDH1* mutation status (the Agilent platform). (D) The correlations of *NMI* methylation levels (presented as β values) and *NMI* expression for both platforms (upper panel, Affymetrix; lower panel, Agilent) were analyzed. (E) *NMI* expression was compared according to status of *PTEN* (left panel) or *EGFR* (right panel) mutation as indicated. Statistical differences were determined by two tailed student's t-test. *, $P<0.05$; **, $P<0.01$; ***, $P<0.001$; ns, not significant. (F) Kaplan-Meier plots were estimated according to different *NMI* gene expression for overall survival (upper panels) and progression-free survival (lower panels) of all GBM patients (left panels), or considering the mutation status of *PTEN* (center panels) or *EGFR* (right panels) simultaneously, in the TCGA cohort (the Agilent platform). *P* values were obtained from log-rank test.

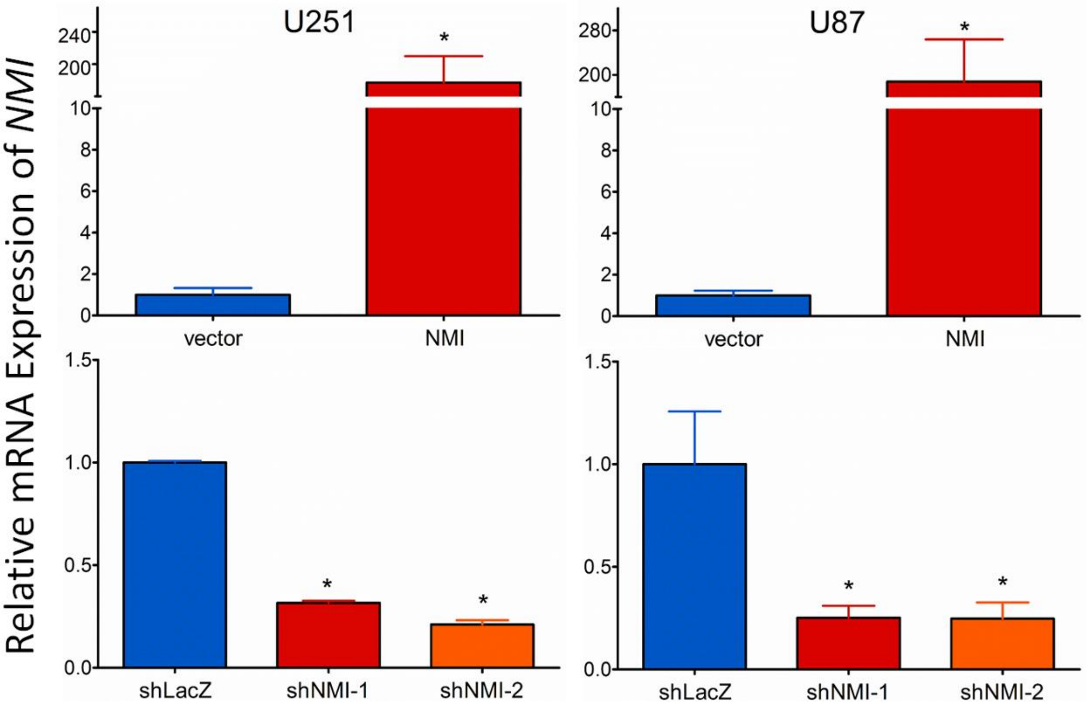

Supplementary Figure S2: Overexpression and knockdown of *NMI* were validated at mRNA level. *NMI* was overexpressed (upper panels) or knocked down (lower panels) by lentiviruses carrying corresponding expression vectors or shRNAs in U251 (left panels) or U87 (right panels) human glioma cell lines. RNA was extracted and subjected to real-time PCR assay. *GAPDH* was used as an internal control. Data are presented as mean ± SEM. Statistical analysis was determined by two tailed student's t-test. *, P<0.05.

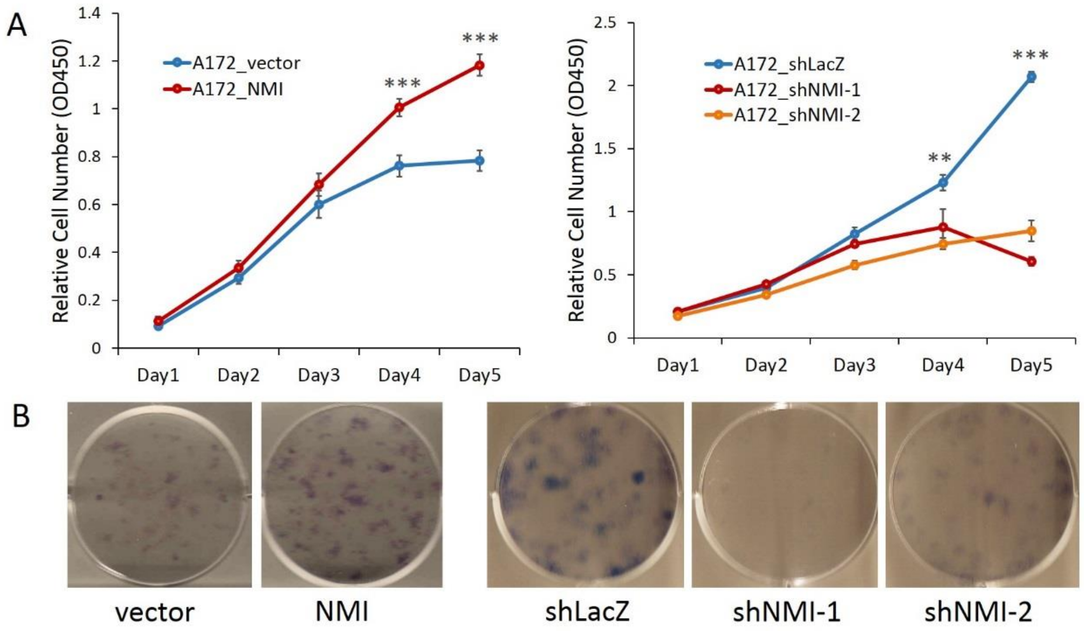

Supplementary Figure S3: NMI promoted A172 glioma cell growth. (A) The cell growth curve of NMI overexpressed (left panel) and silenced (right panel) A172 cells was determined by CCK-8 assay. (B) The long-term proliferation ability of NMI overexpression and knockdown cells was examined using clonogenic cell survival assay. Error bars represent the SEM of the mean value. Statistical analysis was determined by two tailed student's t-test. *, $P<0.05$; **, $P<0.01$; ***, $P<0.001$.

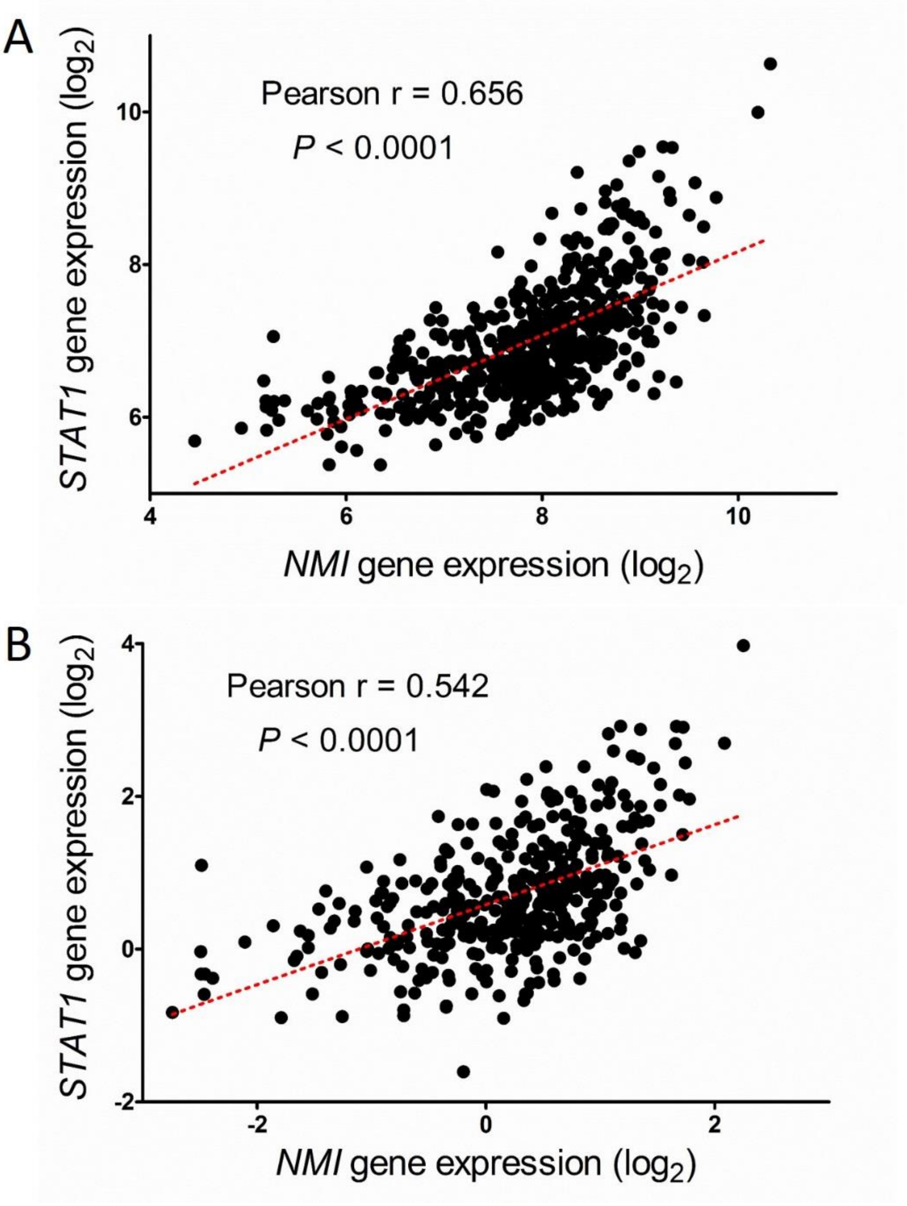

Supplementary Figure S4: The mRNA expression of *NMI* and *TCTN1* for each GBM patient of the TCGA cohort (A, the Affymetrix platform; B, the Agilent platform) was shown in scatter plots and the Pearson r and *P* value of the correlation were indicated. The dashed red line was fit by a linear regression.

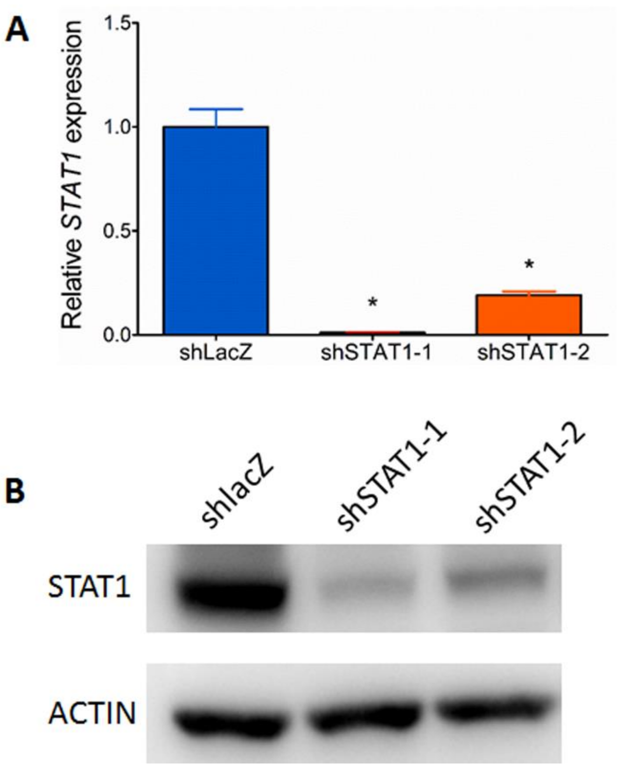

Supplementary Figure S5: Knockdown of *STAT1* was validated at mRNA and protein levels. *STAT1* was knocked down by lentiviruses carrying two independent shRNAs against it, and the efficiency was validated at mRNA level by real-time PCR (A) and protein level by Western blot assays (B). *GAPDH* was used as an internal control for real time PCR assays and ACTIN as a loading control for Western blot assays. Data are presented as mean ± SEM. Statistical analysis was determined by two tailed student's t-test. *, P<0.05.

Supplementary Table S1: Comparison of *NMI* expression according to molecular features of GBM in the TCGA cohort of both platforms

Molecular features		Affymetrix			Agilent		
		N	mean ± SD (log_2)	*P*	N	mean ± SD (log_2)	*P*
G-CIMP	-	469	7.99 ± 0.78	**4.55E-7**	357	0.36 ± 0.67	**9.66E-7**
	+	41	6.96 ± 1.09		29	-0.73 ± 0.93	
IDH1	wild type	372	8.04 ± 0.79	**1.95E-4**	259	0.42 ± 0.69	**5.18E-5**
	mutation	30	7.07 ± 1.24		18	-0.90 ± 1.04	
PTEN	wild type	81	7.55 ± 1.04	**0.001**	46	-0.22 ± 0.97	**3.36E-4**
	deletion	439	7.96 ± 0.81		342	0.35 ± 0.69	
PDGFRA	wild type	405	7.96 ± 0.84	**0.002**	296	0.35 ± 0.71	**0.006**
	amplification	101	7.68 ± 0.85		78	0.09 ± 0.80	
RB1	wild type	227	8.03 ± 0.78	**0.009**	155	0.41 ± 0.74	**0.041**
	mutation	20	8.50 ± 0.55		11	0.88 ± 0.46	
PARK2	wild type	369	7.86 ± 0.84	**0.012**	267	0.26 ± 0.73	0.103
	deletion	136	8.07 ± 0.87		107	0.40 ± 0.73	
EGFR	wild type	70	7.60 ± 1.10	**0.014**	41	-0.17 ± 0.85	**0.32E-4**
	amplification	448	7.94 ± 0.81		344	0.34 ± 0.72	
RB1	wild type	326	7.83 ± 0.87	**0.016**	241	0.27 ± 0.73	0.640
	deletion	193	8.02 ± 0.84		149	0.30 ± 0.79	
CDK6	wild type	442	7.93 ± 0.84	**0.017**	72	0.17 ± 0.83	0.158
	amplification	66	7.66 ± 0.90		318	0.31 ± 0.73	
PIK3CA	wild type	221	8.04 ± 0.79	**0.048**	151	0.44 ± 0.75	0.825
	mutation	26	8.36 ± 0.58		15	0.48 ± 0.51	
TP53	wild type	176	8.13 ± 0.76	**0.049**	116	0.57 ± 0.68	**4.67E-4**
	mutation	71	7.92 ± 0.81		50	0.14 ± 0.77	
MGMT	unmethylated	177	8.08 ± 0.84	0.070	128	0.42 ± 0.78	**0.017**
	methylated	170	7.91 ± 0.89		125	0.19 ± 0.78	

Abbreviations: G-CIMP, Glioma-CpG Island Methylator Phenotype.

Supplementary Table S2: Comparison of Overall survival (OS) and Progression-free survival (PFS) by Kaplan-Meier method according to different *NMI* expression stratified by molecular features of GBM in the TCGA cohort (the Affymetrix platform)

Molecular features		OS		PFS	
		N (*NMI* low/high)	log-rank *P*	N (*NMI* low/high)	log-rank *P*
G-CIMP	-	115/352	0.273	75/226	0.577
	+	29/12	0.352	21/5	0.255
IDH1	wild type	87/284	0.074	58/172	0.154
	mutation	18/11	0.667	11/5	0.506
PTEN	wild type	33/44	**1.90E-04**	21/25	**0.041**
	deletion	109/324	0.316	75/209	0.125
PDGFRA	wild type	94/301	**0.002**	61/193	**0.004**
	amplification	41/60	0.275	29/37	0.637
RB1	wild type	49/170	0.123	30/88	0.535
	mutation	1/18	0.189	1/9	0.636
PARK2	wild type	107/254	**0.001**	69/161	**0.013**
	deletion	29/106	0.531	22/67	0.271
EGFR	wild type	27/39	**3.21E-04**	17/20	**0.001**
	amplification	113/329	0.189	79/214	0.481
RB1	wild type	92/231	**0.030**	58/149	0.201
	deletion	52/138	**0.004**	39/86	**0.005**
CDK6	wild type	34/71	**2.82E-04**	23/42	**0.001**
	amplification	110/298	0.111	74/193	0.354
PIK3CA	wild type	49/164	0.375	30/28	0.280
	mutation	1/24	0.401	1/15	0.634
TP53	wild type	30/138	0.999	20/72	0.189
	mutation	20/50	0.178	11/25	0.105
MGMT	unmethylated	35/141	0.069	26/85	0.443
	methylated	51/118	0.057	25/70	0.223

Abbreviations: OS, overall survival; PFS, progression-free survival; G-CIMP, Glioma-CpG Island Methylator Phenotype.

Supplementary Table S3: Comparison of Overall survival (OS) and Progression-free survival (PFS) by Kaplan-Meier method according to different *NMI* expression stratified by molecular features of GBM in the TCGA cohort (the Agilent platform)

Molecular features		OS		PFS	
		N (*NMI* low/high)	log-rank *P*	N (*NMI* low/high)	log-rank *P*
G-CIMP	-	56/300	0.460	37/187	0.177
	+	20/9	0.822	13/4	0.951
IDH1	wild type	37/221	0.230	25/126	**0.042**
	mutation	14/4	0.907	7/1	0.892
PTEN	wild type	19/27	0.068	12/14	0.295
	deletion	55/285	0.318	37/181	0.068
PDGFRA	wild type	46/248	0.142	28/160	**0.029**
	amplification	21/57	0.241	15/31	0.380
RB1	wild type	21/132	0.506	12/69	0.373
	mutation	0/11	N.A.	0/5	N.A.
PARK2	wild type	52/214	**0.049**	32/133	**0.039**
	deletion	17/89	0.130	14/54	0.085
EGFR	wild type	16/25	**0.012**	10/10	**0.014**
	amplification	57/285	0.348	39/184	0.638
RB1	wild type	41/198	0.122	24/124	0.138
	deletion	34/115	**0.034**	26/71	**0.028**
CDK6	wild type	19/53	**0.002**	12/32	**0.001**
	amplification	56/260	0.432	38/163	0.493
PIK3CA	wild type	20/130	0.632	12/66	0.247
	mutation	1/13	0.601	0/8	N.A.
TP53	wild type	10/104	0.160	8/56	0.460
	mutation	11/39	0.227	4/18	0.499
MGMT	unmethylated	18/109	0.900	14/64	0.745
	methylated	32/93	**0.008**	18/45	0.309

Abbreviations: OS, overall survival; PFS, progression-free survival; G-CIMP, Glioma-CpG Island Methylator Phenotype. N.A., not available due to all patients with corresponding mutation expressed high level of *NMI*.

Supplementary Table S4: Multivariate Cox regression of *NMI* expression for overall survival and progression-free survival in GBM patients of the TCGA cohort (the Agilent platform)

Characteristics [a]	OS		PFS	
	P	HR (95% CI)	*P*	HR (95% CI)
Age (≥60 vs. <60)	**2.08E-4**	2.07(1.41-3.03)	0.277	
MGMT (methylated vs. unmethylated)	0.429		**0.031**	0.62(0.40-0.96)
G-CIMP (positive vs. negative)	0.934		0.114	
Subtype (proneural vs. non-proneural)	**0.036**	1.69(1.03-2.75)	**0.002**	2.66(1.43-4.95)
PTEN (deletion vs. wild type)	0.323		0.423	
EGFR (amplification vs. wild type)	0.152		0.232	
CDK6 (amplification vs. wild type)	0.110		0.106	
IDH1 (mutation vs. wild type)	0.941		0.071	
NMI expression (high vs. low)	**0.034**	1.78(1.05-3.04)	**0.017**	2.10(1.14-3.86)

Abbreviations: G-CIMP, Glioma-CpG Island Methylator Phenotype; OS, overall survival; PFS, progression-free survival; HR, hazard ratio; CI, confidence interval.

[a] Tumor origin was not included in Cox regression since almost all the samples were primary GBM for the Agilent data.

Supplementary Table S5: Sequences of primers and oligonucleotides used for real-time PCR, plasmid construction and shRNAs

Primer name	sense (5'-3')	antisense (5'-3')
Primers for real-time PCR		
NMI-qPCR	CGCGTGGACTATGACAGACA	CAGTAACTCTATGGCAGGTTTGA
STAT1-qPCR	TTGGCACCTAACGTGCTGT	AGTTCGTACCACTGAGACATCCT
GAPDH-qPCR	AGCCACATCGCTCAGACAC	GCCCAATACGACCAAATCC
Primers for plasmid construction		
GFP-NMI	CCGCTCGAGATGGAAGCTGATAAAGATGACACAC	CGGGATCCCTATTCTTCAAAGTATGCTATGTGAGGT
Flag-NMI	GCTCTAGAATGGATTACAAGGATGACGACGATAAGAG ACTCGAGATGGAAGCTGATAAA	CGGGATCCCTATTCTTCAAAGTATGCTATGTGAGGT
pCDH-STAT1-V1	CTAGCTAGCGGCAGGATGTCTCAGTGGTACG	CGCGGATCCGAAAACTGTCGCCAGAGAAGATGA
pCDH-STAT1-V2	CTAGCTAGCGGCAGGATGTCTCAGTGGTACG	CGCGGATCCGAGGTTTGTAAACATGTCACTCTTCTG
mCherry-STAT1-V1	GGAAGATCTGGAGGTGGAGGTATGTCTCAGTGGTACG	CGGGGTACCGAAAACTGTCGCCAGAGAAGATGA
mCherry-STAT1-V2	GGAAGATCTGGAGGTGGAGGTATGTCTCAGTGGTACG	CGGGGTACCGAGGTTTGTAAACATGTCACTCTTCTG
Oligonucleotides for shRNAs		
shLacZ	TGTTCAAGAGATTTAATCAGCGACTGATCCTTTTTTCG ATCAGTCGCTGATTAAA	TCGAGAAAAAAGGATCAGTCGCTGATTAAATCTCTT GAATTTAATCAGCGACTGATCCA
shNMI-1	TGCCAAGCCAGTTCCATTAAATTTCAAGAGAATTTAAT GGAACTGGCTTGGCTTTTTTC	TCGAGAAAAAAGCCAAGCCAGTTCCATTAAATTCTC TTGAAATTTAATGGAACTGGCTTGGCA
shNMI-2	TGTTAACCCGGATTACTGTAAATTTCAAGAGAATTTAC AGTAATCCGGGTTAACTTTTTTC	TCGAGAAAAAAGTTAACCCGGATTACTGTAAATTCT CTTGAAATTTACAGTAATCCGGGTTAACA
shSTAT1-1	TGCCCTGAAGTATCTGTATCCAATTCAAGAGATTGGAT ACAGATACTTCAGGGCTTTTTTC	TCGAGAAAAAAGCCCTGAAGTATCTGTATCCAATCT CTTGAATTGGATACAGATACTTCAGGGCA
shSTAT1-2	TGCTGGAAGATTTACAAGATGAATTCAAGAGATTCATC TTGTAAATCTTCCAGCTTTTTTC	TCGAGAAAAAAGCTGGAAGATTTACAAGATGAATC TCTTGAATTCATCTTGTAAATCTTCCAGCA
